# Avoiding inferior clusterings with misspecified Gaussian mixture models

**DOI:** 10.1038/s41598-023-44608-3

**Published:** 2023-11-06

**Authors:** Siva Rajesh Kasa, Vaibhav Rajan

**Affiliations:** School of Computing, National University of Singapore, COM1, 13, Computing Dr, Singapore, 117417 Singapore

**Keywords:** Computational science, Statistics

## Abstract

Clustering is a fundamental tool for exploratory data analysis, and is ubiquitous across scientific disciplines. Gaussian Mixture Model (GMM) is a popular probabilistic and interpretable model for clustering. In many practical settings, the true data distribution, which is unknown, may be non-Gaussian and may be contaminated by noise or outliers. In such cases, clustering may still be done with a misspecified GMM. However, this may lead to incorrect classification of the underlying subpopulations. In this paper, we identify and characterize the problem of inferior clustering solutions. Similar to well-known spurious solutions, these inferior solutions have high likelihood and poor cluster interpretation; however, they differ from spurious solutions in other characteristics, such as asymmetry in the fitted components. We theoretically analyze this asymmetry and its relation to misspecification. We propose a new penalty term that is designed to avoid both inferior and spurious solutions. Using this penalty term, we develop a new model selection criterion and a new GMM-based clustering algorithm, SIA. We empirically demonstrate that, in cases of misspecification, SIA avoids inferior solutions and outperforms previous GMM-based clustering methods.

## Introduction

The well-established paradigm of model-based clustering assumes data to be generated by a finite mixture model where each component represents a cluster. Gaussian Mixture Models (GMM), in particular, are widely used in a variety of applications^1^. Expectation Maximization (EM) and its variants are, by far, the most popular methods to obtain Maximum Likelihood Estimates (MLE) of GMM parameters^2,3^.

The one-to-one correspondence between fitted components and clusters, that makes model-based clustering appealing, assumes that the underlying model is correctly specified and each data cluster can be viewed as a sample from a mixture component. In real data, the true distribution is rarely known and further, data may be contaminated by noise or outliers from a distribution different from the assumed model. In such *misspecified* settings, MLE may fail to recover the underlying cluster structure^4^.

The behaviour of EM for misspecified GMMs was recently studied by^5,6^ who theoretically quantify the bias in estimates under univariate settings and specific cases, e.g., under- and over-specified number of components. They also characterize the convergence of EM iterates, which, for misspecified GMMs, converge to the Kullback-Leibler projection of the data-generating distribution onto the fitted model class, instead of approximating the true model parameters. Others have studied misspecifcation in the Bayesian setting, e.g., to find a modified likelihood that is robust to mild perturbations from the model^7,8^, to find the number of components^9,10^ and to find identifiability conditions and consistency guarantees^11^. In this work, we study misspecified GMMs in a frequentist parametric setting, with the practical aim of improving clustering accuracy, i.e., inferring correct labels.

It is well known that MLE, even in the absence of misspecification, may give rise to spurious solutions that are local maximizers of the likelihood but without adequate interpretability of cluster structure^12,13^. It is a consequence of the unboundedness of the GMM likelihood function for unrestricted component covariance matrices^14^, that results in solutions from EM with fitted ‘degenerate’ component(s) having very small variance corresponding to a cluster containing very few closely located data points; in the case of multivariate data, there are components with very small generalized variance, lying in a lower-dimensional subspace and close to the boundary of the parameter space^15^. Previous approaches to avoid spurious solutions include multiple restarts^15^ and restricting the parameter space in EM to avoid degeneracy^12,16–18^.

Contamination through noise or outliers, which may be from a population different from the assumed model, may also lead to spurious solutions. Approaches to fit contaminated mixtures include trimming and restricting the parameter space^13,19,20^. In addition, constrained GMMs have been proposed to tackle spurious solutions and contamination^21–24^; typical constraints enforce varying degrees of homoscedasticity across the component covariance matrices in terms of volume, orientation and/or shape. Such constrained models reduce the number of parameters to be estimated, and thereby enable efficient implementations, e.g., in Mclust^25^. However, imposing such hard modeling constraints may be too stringent and can degrade clustering performance as they do not take into account feature dependencies that can vary across the clusters, and may lead to incorrect orientations or shapes of the inferred clusters. As a result, pre-determined constraints on means or covariance matrices are discouraged and a more data-dependent discovery of cluster structure is recommended, e.g., by Zhou et al. ^26^ and Fop et al. ^27^.

In a different line of work, that, to our knowledge, has not studied misspecification, gradient-based methods for MLE of GMMs have been investigated^28–31^. In general, EM has numerous advantages over gradient-based methods as discussed in Xu and Jordan ^32^. More recently, there has been considerable development of Automatic Differentiation for Gradient Descent (AD–GD),

that obviate the need to derive closed-form expressions of gradients and thereby facilitate inference in complex models such as deep neural networks as well as mixture models^33,34^.

In our study, we first compare the clustering performance—in terms of Adjusted Rand Index (ARI)^35^—of GD and EM with misspecified GMMs. Our simulations reveal a previously unreported class of poor clustering solutions, with both EM and GD. These solutions, that we call *inferior*, occur frequently in MLE procedures (irrespective of the initialization), have asymmetric fitted covariances that vary in their sizes and have poor cluster interpretability. Thus, they differ in their characteristics from spurious solutions. More details are given in section "Inferior clustering solutions". Our theoretical analysis on a specific setting of univariate mixtures and under-specified number of components yields evidence on the connection between asymmetry of fitted components and misspecification. It also motivates the design of a new penalty term, based on the Kullback Leibler divergence between pairs of fitted component Gaussians, to avoid inferior clusterings. Closed forms for the gradients of this penalized likelihood are difficult to derive and we leverage AD-GD to develop algorithms

for clustering (Sequential Initialization Algorithm aka SIA) and a model selection criterion (**M**aximum absolute **P**airwise difference between **KL** divergence values aka MPKL). Our functional regularization approach, that enforces soft constraints tunable by varying the hyperparameters, avoids both spurious solutions and shortcomings of imposing hard modeling constraints. Our extensive experiments demonstrate the advantage of our methods in clustering with misspecified GMMs.

To summarize, our contributions in this paper are:

- We conduct an empirical analysis to compare the clustering performance of GD and EM inference on unconstrained and misspecified GMMs.
- We identify and characterize the problem of *inferior* clusterings that have high likelihood and low ARI, similar to spurious solutions, in both EM and GD. Unlike spurious solutions, these solutions have fitted components that are markedly asymmetric with respect to their orientation and sizes, and occur frequently with many different initializations. We theoretically examine how asymmetry of fitted components varies with misspecification.
- We propose a new penalty term that is designed to avoid inferior solutions and prove that the penalized likelihood is bounded and hence avoids degeneracy. Using this penalty term, we develop MPKL, a model selection criterion and SIA, an AD-GD based clustering algorithm.
- Experiments on synthetic and real datasets demonstrate the advantages of SIA, over previous clustering methods with both constrained and unconstrained GMMs, in cases of misspecification.

## Background

Let $f({\varvec{x}}; {\theta })$ be the density of a *K*-component mixture model. Let $f_k$ denote the $k\textrm{th}$ component density with parameters $\theta _k$ and weight $\pi _k$. The density of the mixture model is given by $f({\varvec{x}} ; {\theta }) =\sum _{k=1}^{K} \pi _{k} f_{k}\left( {\varvec{x}} ; {\theta }_{k}\right) ,$ where $\sum _{k=1}^{K} \pi _k = 1$ and $\pi _k \ge 0$ for $k = 1,\ldots , K$ and $\theta$ denotes the complete set of parameters. In a GMM, each individual component $f_k$ is modeled using a multivariate Gaussian distribution ${\mathcal {N}}(\mu _k, \Sigma _k)$ where $\mu _k$ and $\Sigma _k$ are its mean and covariance respectively. Appendix A lists all the symbols used herein.

Given *n* independent and identically distributed (*iid*) instances of *p*-dimensional data, $[x_{ij}]_{n \times p}$ where index *i* is used for observation, and *j* is used for dimension, Maximum Likelihood Estimation (MLE) aims to find parameter estimates $\hat{\theta }$ from the overall parameter space $\Theta$ of $f(\theta )$ such that probability of observing the data samples ${{\textbf {x}}}_1, \dots , {{\textbf {x}}}_n$ is maximized, i.e., ${\hat{\theta }}=\mathop {\mathrm {arg\,max}}\limits _{\theta \in \Theta } {\mathcal {L}}(\theta )$, where, $\mathcal {L(\theta )} = \frac{1}{n}\sum _i \log f({{\textbf {x}}}_i;\theta )$ is the empirical expected loglikelihood.

Following^36^, if the observed data are *n* *iid* samples from a probability distribution $P(\eta ^*)$ (where $\eta ^*$ denotes the *true* set of parameters) and the fitted model has the same functional form *P*(.), then the model is said to be correctly specified. Otherwise, the model is said to be *misspecified*. Note that when the number of dimensions are greater than the number of datapoints ($p>n$), there are not enough datapoints to determine if the fitted model and data-generating distribution are parameterized by the same model, even along a single dimension and so, the notion of misspecification is moot. Appendices B and C give an overview of, respectively, spurious solutions and likelihood-based model selection criteria (such as Akaike Information Criterion (AIC) and Bayesian Information Criterion (BIC)).

Recent reviews on Automatic Differentiation (AD) can be found in^37,38^; a brief review is in Appendix D. To obtain MLE of GMMs, EM elegantly solves 3 problems: (1) Intractability of evaluating the closed-forms of the derivatives, (2) Ensuring positive definiteness of the covariance estimates ${\hat{\Sigma }}_k$, and (3) Ensuring the constraint on the component weights ($\sum _k {\hat{\pi }}_k = 1$). *Problem 1* is inherently solved by the use of AD. *Problems 2* can be addressed through simple reparametrizations in the form of matrix decomposition^34,39^. *Problems 3* can be addresed through the unbounded $\alpha _k$ as the log-proportions where $\log \pi _k = \alpha _k - {\log (\sum _{k^{'}=1}^K e^{\alpha _{k^{'}}})}$. Details are in Appendix D. Constraints used in EM to mitigate spurious solutions and contamination can also be used in AD-GD through changes in the update equations.

## Inferior clustering solutions

In this section, we illustrate inferior clustering solutions through an empirical comparison of EM and AD-GD in cases of misspecification. We also theoretically analyze the role of asymmetric fitted components in misspecified Gaussian mixture models.

### Simulation study

**Figure 1:**
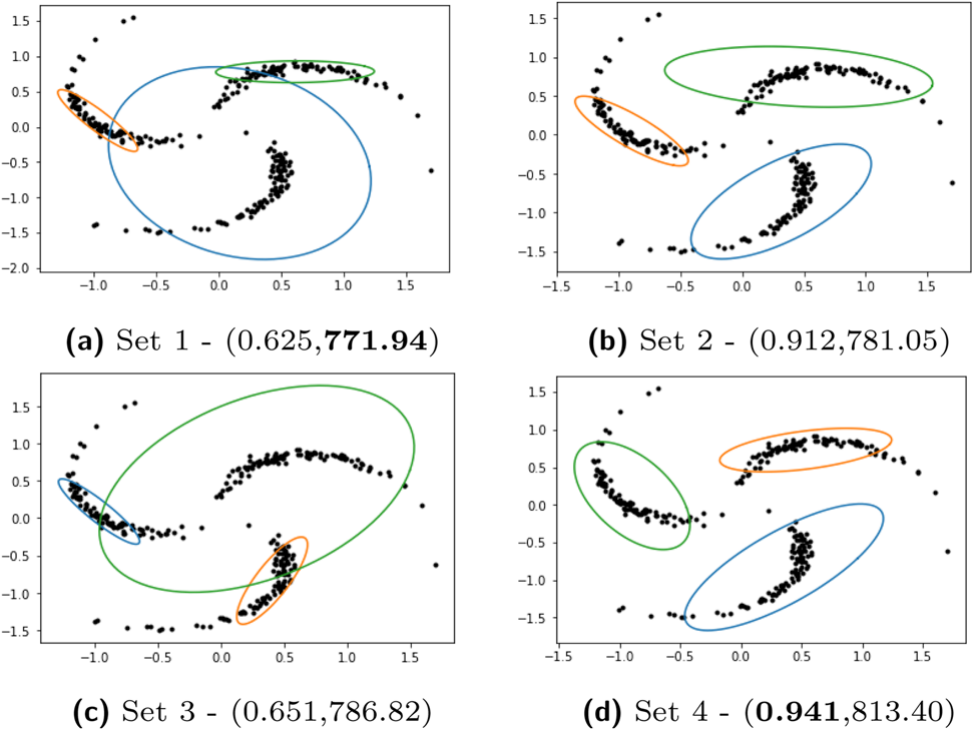
4 clustering solutions obtained with AD-GD using different initializations on Pinwheel data; Sets refer to groups in Table 1; in parentheses: (ARI, AIC), best values in bold.

We study the clustering solutions obtained by fitting misspecified GMM on Pinwheel data, also known as warped GMM, with 3 components and 2 dimensions^40^. Pinwheel data is generated by sampling from Gaussian distributions, and then stretching and rotating the data. The centers are equidistant around the unit circle. The variance is controlled by parameters *r*, the radial standard deviation and *t*, the tangential standard deviation. The warping is controlled by a third parameter, *s*, the rate parameter. Thus, the extent of misspecification (i.e., the deviation of the data from the assumed Gaussian distributions in GMM) can be controlled using these parameters. An example is shown in Fig. 1. We generate 1800 Pinwheel datasets with different combinations of parameters. In addition, we also simulate 1800 3-component, 2-dimensional datasets from GMM with varying overlap of components (to analyze as a control case where there is no misspecification in fitting a GMM). For each dataset we obtain two clustering solutions, one each using EM and AD-GD. We run both the algorithms till convergence, using the same initialization and stopping criterion. We use ARI to evaluate performance where higher values indicate better clustering. Details are in Appendix E.

Our experiments on these 3600 datasets show that EM outperforms AD-GD in both cases—when there is misspecification and no missspecification. However, when there is misspecification, we find that for both EM and AD-GD, there are many *inferior* solutions that have low ARI and unequal fitted covariances, often with one fitted component having relatively large covariance, resulting in a high degree of overlap between components. We illustrate this using Pinwheel data generated with parameters $r=0.3$, $t=0.05$ and $s=0.4$. Both EM and AD-GD are run with 100 different initializations. We group the solutions into 4 sets based on AIC as shown in Table 1, which also shows the average AIC and ARI obtained by AD-GD and the number of EM and AD-GD solutions in each set. Table 27 (in Appendix E) show the statistics for EM solutions which are very similar. We visualize the clustering for one solution from each of these sets in Fig. 1.

**Table 1:** Summary statistics of clustering solutions over 100 random initializations on the Pinwheel dataset (see Fig. 1), grouped into 4 sets based on AIC ranges.

Set	AIC	AD-GD								# Solutions	
	Range	AIC	ARI	$\pi _1$	$\pi _2$	$\pi _3$	$|\Sigma _1|$	$|\Sigma _2|$	$|\Sigma _3|$	EM	AD-GD
1	771–773	771.9 (6e−8)	0.625 (2e−16)	0.257 (3e−6)	0.265 (3e−6)	0.477 (1e−6)	0.0002 (1e−9)	0.0005 (4e−9)	0.123 (4e−7)	24	19
2	781–782	781.1 (3e−6)	0.912 (0)	0.306 (1e−5)	0.341 (3e−5)	0.352 (1e−5)	7e−4 (5e−9)	0.01 (5e−9)	0.01 (5e−7)	4	3
3	786–787	786.8 (2e−7)	0.652 (0)	0.257 (5e−6)	0.267 (3e−6)	0.475 (3e−6)	2e−4 3e−9	5e−4 (3e−9)	0.156 (2e−6)	16	27
4	788–850	815.0 (17.84)	0.806 (0.06)	0.28 (0.015)	0.315 (0.010)	0.403 (0.024)	6e−4 (4e−4)	3e−3 (1e−3)	0.047 (0.018)	56	51

Mean (Standard Deviation) of parameter estimates from AD-GD, EM estimates (in Appendix E) are similar.

We observe that both EM and AD-GD obtain very similar solutions in terms of AIC and ARI for this dataset. The best *average* ARI is that of set 2 (row 2 of Table 1). These solutions are obtained in less than 5% of the cases. The *overall best* ARI, of 0.941, is from a solution in set 4 that has a high AIC value of 813.4 as shown in Fig. 1d. The best AIC, of 771.9, is obtained by a solution from set 1 which has considerably lower ARI of 0.625 as shown in Fig. 1a. Thus, we see solutions with high likelihood and low ARI. We observe that there are many such inferior solutions in sets 1, 3 and 4 having a fitted component with large variance, also seen in the specific solutions in Fig. 1.

In cases of misspecification, inferior clusterings from both EM and AD-GD, are found to have fitted covariances that differ considerably in their orientations and sizes. We observe this in the solutions in Fig. 1 and through the summary statistics in Table 1. We find that such inferior solutions in misspecified models occur frequently with many different initializations, and typically when there is a component with large variance. This is different from the characterization of spurious solutions (see Appendix B) that are found to occur rarely, only with certain initializations and due to a component with small variance^1^.

We find similar inferior solutions with low ARI and low AIC in cases where Gaussian components are contaminated by a Student’s-t distribution and random noise (details in Appendix F). Further, we have observed similar effects of misspecification in higher dimensions as well. Illustrations in datasets of up to 100 dimensions are in Appendix F.

### Misspecification and asymmetric components

We now theoretically examine how asymmetry of fitted components varies with misspecification in the case of univariate mixtures, following the setting of^5,6,41^.

**Figure 2:**
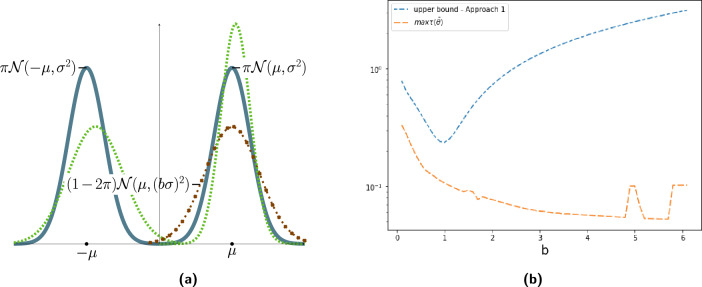
(**a**) True distribution is a GMM  and a contamination . We fit a 2-component (misspecified) GMM . (**b**) Empirical comparison of $\tau (\bar{\theta })$ and its upper bound with varying *b*.

Let the true data generating distribution be $G^{*} = \pi {\mathcal {N}}(-\mu , \sigma ^2) + \pi {\mathcal {N}}(\mu , \sigma ^2) + (1-2\pi ) {\mathcal {N}}(\mu ,b^2\sigma ^2)$, with $0 < \pi \le 0.5$. Without loss of generality, we assume $\mu > 0$. When $\pi = 0.5$, the true distribution is a symmetric 2-component GMM, with the component means equidistant on either side of the real line as shown in Fig. 2a. In this case, it has been shown that under some identifiability conditions, fitting a 2-component GMM (i.e., without any misspecification) on the data sampled from the true distribution using MLE leads to symmetric fitted components whose parameters converge to that of the true distribution^32,36^. As $\pi$ is reduced from 0.5, an additional Gaussian component with mean $\mu$ (that coincides with one of the component means) and variance $b^2\sigma ^2$ is introduced. Fitting a 2-component GMM in the case when $\pi < 0.5$ leads to a misspecified model. We analyze the asymmetry of the fitted components for $\pi < 0.5$ with varying misspecification (*b*).

Let the fitted misspecified distribution be $G^{\prime }(\bar{\theta }) = (1-\bar{\pi }){\mathcal {N}}(\bar{\mu }_1,\bar{\sigma }^2_1) + \bar{\pi } {\mathcal {N}}(\bar{\mu }_2,\bar{\sigma }^2_2)$, where $\bar{\theta } = (\bar{\mu }_1,\bar{\sigma }_1^2,\bar{\mu }_2,\bar{\sigma }_2^2,\bar{\pi })$ and $\bar{\theta } \in \arg \min _{\theta \in \Theta } \textrm{KL}\left( {G_{*}}, {G^{\prime }(\theta )}\right)$. Note that this is a projection to the fitted model and is the best possible estimator^5^. Let ${\text {erf}}$ be the Gauss error function^42^ which is an odd function, whose values lies in $(-1,1)$ and is related to the CDF of a standard normal distribution as $\varvec{\Phi }(x) = \frac{1}{2} \left[ 1+{\text {erf}}\left( \frac{x}{\sqrt{2}}\right) \right]$.

When misspecification is small, the means of fitted components $\bar{\mu }_1,\bar{\mu }_2$ typically have opposite signs, as the true components flank the y-axis. When there is misspecification, we expect ${\text {erf}}\left( \frac{\bar{\mu }_1}{\sqrt{2}\bar{\sigma }_1 } \right)$ and ${\text {erf}}\left( \frac{\bar{\mu }_2}{\sqrt{2}\bar{\sigma }_2 } \right)$ to have unequal and opposite signs. We define an asymmetry coefficient:

$$\begin{aligned} \tau (\bar{\theta }) = (\bar{\pi }) {\text {erf}}(\frac{\bar{\mu }_2}{{\sqrt{2}\bar{\sigma }_2}}) + (1-\bar{\pi }) {\text {erf}}(\frac{\bar{\mu }_1}{{\sqrt{2}\bar{\sigma }_1}}). \end{aligned}$$

$\tau (\bar{\theta })$ measures a form of asymmetry in $\frac{\mu }{\sigma }$ of the fitted components when there is misspecification. When there is no misspecification, since MLE estimates converge to the true parameters asymptotically, $\tau (\bar{\theta })$ also converges to zero. However, when there is misspecification, the variance of one of the components, say $\sigma _1$, tends to be much larger than that of the other component (as seen in Table 1); in such cases, ${\text {erf}}\left( \frac{\mu _1}{\sqrt{2}\sigma _1} \right)$ would be much closer to zero compared to the error function for the other component and $\tau (\bar{\theta })$ would converge to a non-zero value. We derive bounds on $\tau (\bar{\theta })$ as follows (proof in Appendix G):

#### Theorem 1

Let the true data generating distribution be $G^{*} = \pi {\mathcal {N}}(-\mu , \sigma ^2) + \pi {\mathcal {N}}(\mu , \sigma ^2) + (1-2\pi ) {\mathcal {N}}(\mu ,b^2\sigma ^2)$, with $0 < \pi \le 0.5$. Let the fitted misspecified distribution be $G^{\prime }(\bar{\theta }) = (1-\bar{\pi }){\mathcal {N}}(\bar{\mu }_1,\bar{\sigma }^2_1) + \bar{\pi } {\mathcal {N}}(\bar{\mu }_2,\bar{\sigma }^2_2)$, with asymmetry coefficient $\tau (\bar{\theta })$, where $\bar{\theta } = (\bar{\mu }_1,\bar{\sigma }_1^2,\bar{\mu }_2,\bar{\sigma }_2^2,\bar{\pi })$ and $\bar{\theta } \in \arg \min _{\theta \in \Theta } \textrm{KL}\left( {G^{*}}, {G^{\prime }(\theta )}\right)$. Let $C_2 := (1-2{\pi })(-\log b + 0.5b^2 - 0.5) + {\pi }\frac{2\mu ^2}{\sigma ^2}$ and $C_w := \frac{1-2w}{2} \text {erf}\left( \frac{-\mu }{\sqrt{2}\sigma } \right)$. Then,

$$\begin{aligned} -\sqrt{2C_2} + 2C_w \le \tau (\bar{\theta }) \le \sqrt{2C_2} + 2C_w. \end{aligned}$$

Note that $C_2$ and $C_w$ depend only on the true parameters $(\pi ,\mu ,\sigma ,b)$ and are constants with respect to the fitted model. Assuming that the true parameters are known, these bounds provide a certification on whether the fitted parameters $\bar{\theta }$ indeed correspond to the maximum likelihood, thus helping to filter out fitted parameters corresponding to spurious solutions and undesirable local optima.

We empirically compare the upper bound and observed value of $\tau (\bar{\theta })$ when $\mu =5.0,\sigma =10,\pi =0.35$ by varying the values of *b* (the behaviour of the lower bound is qualitatively similar). For a given value of $b \in \{0.1,0.2,\dots , 6 \}$, we simulate 50 datasets and pick the maximum of $\tau (\bar{\theta })$ among these 50 output solutions. The upper bound and the observed maximum are plotted in Fig. 2b.

At around $b=1$ (when there is no misspecification), the upper bound reaches its minimum. As *b* moves away from 1, the upper bound increases which illustrates that as misspecification increases, the asymmetry in the fitted components also increases. Additional plots for other values of $(\mu ,\sigma ,\pi )$ are in Appendix G. We observe that, if one of the components has a relatively large variance, then many datapoints may be wrongly assigned to this component leading to poor clustering.

## A penalized clustering method

### A new penalty term

Our analysis in the previous section naturally leads to the goal of reducing the asymmetry in the fitted components to improve clustering with misspecified GMMs. To this end, we develop a new functional penalty term that (i) penalizes differences in orientations and sizes of the fitted components to avoid inferior solutions and (ii) bounds the penalized likelihood to avoid spurious solutions during ML estimation.

Our penalty term is based on the KL-divergence between component Gaussians. Let ${\mathcal {N}}_k$ denote the multivariate Gaussian distribution ${\mathcal {N}}(\mu _k,\Sigma _k)$. The KL-divergence $KL\left( {\mathcal {N}}_1, {\mathcal {N}}_2\right)$ is given below where each term can provide useful penalties:

$$\begin{aligned} \small \frac{1}{2} [ \underbrace{\log \frac{|\Sigma _2|}{|\Sigma _1|}}_{A} + \underbrace{\text {tr} \{ \Sigma _2^{-1}\Sigma _1 \} - p}_{B} + \underbrace{(\mu _2 - \mu _1)^T \Sigma _2^{-1}(\mu _2 - \mu _1)}_{C}] \end{aligned}$$

A Penalizes the difference in size of the covariance matrices of the two components. Even if the directions of principal axes of the two covariance matrices are different, this term will be zero if the component determinants are exactly the same, since $\log (1) = 0$.
B Penalizes the difference in orientations, i.e., if the directions of principal axes of the component covariance matrices are vastly different. When $\Sigma _1$ and $\Sigma _2$ are equal, this penalty term becomes zero.
C Penalizes the assignment of a single cluster to faraway outlier points which have an extremely low likelihood of being observed. If some outlier points are assigned a single cluster, then the cluster center, $\mu _1$, would be different from the cluster center $\mu _2$ of non-outlier data, and so, the Mahalonobis distance between the cluster centers, $\Vert \mu _1 - \mu _2 \Vert$, would be high, with higher penalization.

**Figure 3:**
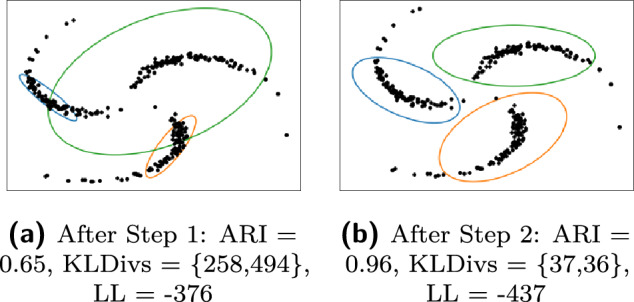
Clustering using SIA: compare with Fig. 1.

KL-divergence is not symmetric about $\Sigma _1$ and $\Sigma _2$. If there is an order of magnitude difference in the covariance matrices $\Sigma _1$ and $\Sigma _2$, it can be detected through the values of $KL\left( {\mathcal {N}}_1, {\mathcal {N}}_2\right)$ and $KL\left( {\mathcal {N}}_2, {\mathcal {N}}_1\right)$. The difference in their values primarily stems from the difference between the terms $(\mu _2 - \mu _1)^T \Sigma _2^{-1}(\mu _2 - \mu _1)$ and $(\mu _2 - \mu _1)^T \Sigma _1^{-1}(\mu _2 - \mu _1)$, and $\text {tr} \{ \Sigma _2^{-1}\Sigma _1 \}$ and $\text {tr} \{ \Sigma _1^{-1}\Sigma _2 \}$. The difference in these two KL divergence values provides signal about the overlap or asymmetry of the two Gaussians. This notion is generalized to a *K*-component GMM, through the combinatorial KL divergences, KLF (forward) and KLB (backward), where $1 \le k_1, k_2 \le K$: $KLF := \sum _{k_1< k_2} KL\left( {\mathcal {N}}_{k_1}, {\mathcal {N}}_{k_2}\right) ; KLB := \sum _{k_2 < k_1} KL\left( {\mathcal {N}}_{k_1}, {\mathcal {N}}_{k_2}\right)$. Well separated equal-sized clusters typically have similar values of KLF and KLB (By well-separated we refer to the case where the means of the Gaussian distributions representing the two clusters are far apart from each other relative to their spreads (as dictated by their respective covariance matrices). In this scenario, the values of both KLF and KLB are both dominated by the term B and term C i.e. the trace term and the Mahalonobis distance term. The difference between KLF and KLB will be mainly due to the differences in terms B, C between the pairwise KL-Divergences. Now consider a scenario, where the spread of one of the cluster is much higher compared to the other one. In this not-well-separated case, the terms B,C will differ more significantly between the pairwise KL-Divergences. This effect will be later seen clearly in Fig. 3 where the KLF and KLB values are high when the clusters not well separated.). We denote both the values by $KLDivs = \{KLF,KLB\}.$ We note that these two sums (KLF + KLB) together give the sum of Jeffrey’s divergence between all components^43^. In the clustering outputs shown in Fig. 1, we see that in solution (c) from set 3, where clustering is poor, KLF$=258$ and KLB$=494$ (the difference is high), while in solution (d) from set 4, which has better clustering, KLF$=127$ and KLB$=64$ (with low difference).

Our proposed penalty term is a weighted sum of the *KLF* and *KLB* terms: $- w_1 \times KLF - w_2 \times KLB$, with negative weights $(-w_1,-w_2)$. With positive weights GD will further shrink the smaller clusters. Negative weights lead to reduced overlap as well as clusters of similar volume. In our experiments, we found that choosing $w_1 = w_2$ works in almost all cases. Further, choosing $w_1 = w_2$ makes the penalized objective invariant to permutation in the component labels. In general, we can say that for all positive values of $w_1,w_2$, the weighted penalty term (made up of KLF, KLB) will be always positive as both KLF and KLB are summation of KL divergences. Alternatively we also take a closer look at the individual terms that make up KLF and KLB. Term C is the Mahalonobis term which will always be positive. Term A+B is known as the *Burg Matrix Divergence* or the *LogDet Divergence*, which is again guaranteed to be positive^44^. However, the same cannot be said for the individual terms A and B separately. E.g., if we were to consider the contribution of the log determinant terms towards the penalty in isolation, it is possible that the aggregate of the log determinant terms can be negative. As a minor side point, we also want to point out that when we set $w_1=w_2$, as we did in our experiments, the log determinant terms cancel out each other in the final expression and thus, only terms B and C would contribute to the penalty.

Optimization of likelihood with these penalty terms can be implemented effortlessly through AD-GD where gradients in closed forms are not required. However, the use of such complex penalties is difficult within EM. We cannot obtain closed forms of the covariance estimates. Closed forms for the mean update can be derived (Appendix H) but is laborious and each mean update depends on means for all other components, and hence cannot be parallelized in EM.

### Sequential initialization algorithm (SIA)

SIA consists of two steps. In Step I of SIA, we use the loglikelihood ${\mathcal {L}}$ as the objective and run EM or AD-GD to fit a GMM. Typically, the output at the end of Step I will have unequal KL-divergences for misspecified models. The parameters at the end of Step I are used to initialize the algorithm in step II where we modify the objective function to:

1 $$\begin{aligned} {\mathcal {M}} = {\mathcal {L}} - w_1 \times KLF - w_2 \times KLB \end{aligned}$$

After the second optimization step the likelihood decreases but the KL-divergence values, KLF and KLB, come closer. The complete algorithm is presented in Algorithm 1.

**Algorithm 1:**
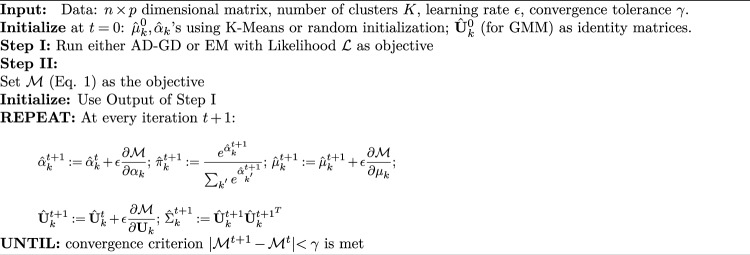
**SIA**.

In SIA, after Step I we know the likelihood and model parameters of the unpenalized fitted model. In Step II, we choose $w_1,w_2$ in such a way that the overall likelihood of the model doesn’t reduce drastically, to find solutions (with less dissimilar covariances) close to the solution after Step I. In our experiments, the values of $w_1,w_2$ have been kept equal and chosen from $\{0,0.25,0.5,1,1.25\}$ using the MPKL criterion (described below).

### SIA objective is bounded

We consider maximum likelihood estimation of parameters $\theta$ of a two-component GMM, from *n* samples, each of *p*-dimensions, using SIA. We prove that our penalized log-likelihood ${\mathcal {M}}$ is bounded, and thus SIA alleviates the problem of degeneracy and spurious solutions in unconstrained GMMs.

#### Theorem 2

Let *C* denote a constant dependent only on $p,n,w_1,w_2, c$ and independent of $\theta$. Assume that the spectral norm of the generalized variances obtained using MLE, $\Sigma _1, \Sigma _2$, is bounded by $c < \infty$ (as a regularity condition) and, without loss of generality, assume $| \Sigma _1| \le | \Sigma _2|$ and that the loglikelihood ${\mathcal {L}} \rightarrow \infty$ when the first component collapses on a datapoint, i.e., $| \Sigma _1| \rightarrow 0$. For non-negative weights $w_1,w_2$, and any iteration *t*, SIA objective function ${\mathcal {M}}$ is always bounded:

$$\begin{aligned} {\mathcal {M}} \le - \frac{{1} }{2} (1+w_2)\left( \log ( |\frac{w_2}{1+w_2} \Sigma _2 |) + p \right) + C. \end{aligned}$$

The proof, in Appendix G, also clarifies that the result generalizes to arbitrary number of components. The bound is given in terms of $w_2$ because we assume $| \Sigma _1| \le | \Sigma _2|$ (the objective is not symmetric with respect to $w_1$ and $w_2$). Hence, $w_1$ does not appear in the theorem statement. Also note that, $w_1$ and $w_2$ are the only controllable hyper-parameters. By setting $w_2 = 0$, we can see that the maximum of unpenalized likelihood is unbounded. If a $\Sigma$ collapses, the log-likelihood may increase but the trace term and the determinant in the penalization will explode. Hence, by having negative weights $-w_1, -w_2$ we can ensure that ${\mathcal {M}}$ is bounded and control the behavior of the algorithm. Note that both $| \Sigma _1|, | \Sigma _2|$ will not collapse to zero in a maximum-likelihood estimation approach because if both collapse the log-likelihood goes to negative infinity (which is easy to verify). A visualization of the likelihood surface, with and without penalty terms, is shown in Appendix I. The fact that there *exists* an upper bound to the penalized likelihood (even when the generalized variance of one of the components collapses to zero), irrespective of its tightness, is sufficient to show that SIA avoids degenerate and spurious solutions.

### MPKL: a model selection criterion

We define the **M**aximum absolute **P**airwise difference between **KL** divergence values (MPKL) for a *K*-component GMM as:

2 $$\begin{aligned} \max _{1 \le k_1,k_2 \le K} | KL\left( {\mathcal {N}}_{k_1}, {\mathcal {N}}_{k_2}\right) - KL\left( {\mathcal {N}}_{k_2}, {\mathcal {N}}_{k_1}\right) | \end{aligned}$$

MPKL is an indicator of how well the clusters are separated. It is invariant to permutation in cluster labels and can be used as a criterion for selecting the number of clusters. For a chosen range of number of clusters ($2,\ldots ,L$), we compute MPKL for each value and choose *K* that minimizes MPKL: $\text {argmin}_{K \in [2,\ldots ,L]} MPKL$. Note that MPKL criterion is independent of SIA and can be potentially useful for any GMM-based method, even in the absence of suspected misspecification. In our experiments (Evaluation on real datasets and Appendix L.2), we show that use of MPKL aids both GD and EM based methods.

### Computational complexity

The computational complexity is dominated by evaluating *KLF* and *KLB* which involves $O(K^2)$ matrix inversion steps ($O(p^3)$). Therefore, the overall time complexity of both SIA and MPKL is $O(K^2p^3)$. This is comparable to most GMM inference methods (e.g., EM) due to the $O(p^3)$ matrix inversion step. Wall-clock times of EM, AD-GD and SIA implementations are compared in Appendix J.

### Illustrative examples

Figure 3 shows the clustering solution (set 3) obtained on the Pinwheel data discussed in Inferior clustering solutions, after steps I and II of SIA. After step II, compared to the clustering after step I, the likelihood decreases, both the KLF and KLB values decrease, the ARI increases, and the clusters have less overlap. The same is observed for the other three sets of clustering solutions. Similar illustrations on datasets contaminated with Student’s-t and random noise are in Appendix F.

**Table 2:** Summary of simulation experiments; *K*: no. of components, *p*: dimensionality, *n*: no. of data points, *r*: ratio of weights, $\mu$: means, $\Sigma$: covariances. In each case we use $p = 2, \ldots , 20$.

	Condition(s) Varied	*K*	$\mu$	$\Sigma$	*r*	*n*	Sections
1	Cluster separation	3	varied	unit	1	300*p*	Varying cluster separation
2	Covariance and mixture weights	2	Fixed	Varied	$\{0.2, 0.5, 1\}$	$\{120p, 150p, 200p\}$	Varying covariance structures and unbalanced mixtures
3	#components	Under-specified	Fixed	Unit	1	150	L.1.1
4	#components	Over-specified	Fixed	Unit	1	100	L.1.2

**Table 3:** ARI (mean and SD) on varying $\lambda$ and $p = 2$.

$\lambda$	3	4	5	7
SIA	0.021 (0.052)	0.073 (0.147)	0.135 (0.270)	0.281 (0.325)
AD-GD	0.021 (0.052)	0.064 (0.146)	0.129 (0.218)	0.278 (0.334)
EM	0.024 (0.042)	0.043 (0.060)	0.111 (0.206)	0.284 (0.355)
Mclust	**0.104** **(0.019)**	**0.189** **(0.038)**	**0.226 ** ** (0.036)**	**0.447** **(0.224)**

Significant values are in bold.

**Table 4:** ARI (mean and SD) on varying $\lambda$ and $p=3$.

$\lambda$	3	4	5	7
SIA	0.119 (0.081)	0.213 (0.090)	**0.399** **(0.099)**	**0.881 ** **(0.069)**
AD-GD	0.105 (0.059)	0.198 (0.073)	0.381 (0.094)	0.873 (0.071)
EM	0.075 (0.038)	0.221 (0.117)	0.395 (0.178)	0.632 (0.358)
Mclust	**0.139** ** (0.018)**	**0.228 ** **(0.041)**	0.290 (0.081)	0.731 (0.228)

Significant values are in bold.

**Table 5:** ARI (mean and SD) on varying $\lambda$ and $p=5$.

$\lambda$	3	4	5	7
SIA	0.163 (0.110)	**0.504** ** (0.129)**	**0.757** **(0.103)**	**0.926** **(0.036)**
AD-GD	0.178 (0.075)	0.451 (0.088)	0.678 (0.089)	0.908 (0.048)
EM	0.143 (0.116)	0.316 (0.195)	0.527 (0.297)	0.856 (0.254)
Mclust	**0.184 ** **(0.013)**	0.321 (0.154)	0.610 (0.262)	0.839 (0.169)

Significant values are in bold.

**Table 6:** ARI (mean and SD) on varying $\lambda$ and $p=10$.

$\lambda$	3	4	5	7
SIA	**0.559** **(0.109)**	**0.836** **(0.130)**	**0.893 ** **(0.239)**	**0.983** **(0.015)**
AD-GD	0.553 (0.109)	0.773 (0.220)	0.846 (0.235)	0.949 (0.038)
EM	0.340 (0.189)	0.477 (0.325)	0.571 (0.412)	0.835 (0.294)
Mclust	0.265 (0.052)	0.626 (0.270)	0.727 (0.259)	0.829 (0.237)

Significant values are in bold.

**Table 7:** ARI (mean and SD) on varying $\lambda$ and $p=20$.

$\lambda$	3	4	5	7
SIA	**0.850 ** **(0.162)**	**0.893 ** **(0.172)**	**0.947 ** **(0.469)**	**0.966** **(0.441)**
AD-GD	0.846 (0.058)	0.843 (0.279)	0.861 (0.491)	0.926 (0.441)
EM	0.684 (0.234)	0.750 (0.345)	0.879 (0.263)	0.995 (0.005)
Mclust	0.328 (0.008)	0.747 (0.256)	0.779 (0.250)	0.796 (0.249)

Significant values are in bold.

**Table 8:** ARI (mean and SD) for different *p* and $N_2 = 20$.

p	2	3	5	10	20
SIA	**0.192 ** **(0.333)**	**0.128** **(0.241)**	**0.077 ** **(0.086)**	**0.048 ** **(0.056)**	0.0311 (0.035)
AD-GD	0.168 (0.328)	0.122 (0.244)	0.020 (0.079)	0.001 (0.007)	0.001 (0.01)
EM	0.162 (0.343)	0.084 (0.234)	0.064 (0.088)	0.0344 (0.054)	0.030 (0.035)
MClust	0.168 (0.329)	0.086 (0.181)	0.052 (0.079)	0.045 (0.028)	**0.035 ** **(0.015)**

Significant values are in bold.

**Table 9:** ARI (mean and SD) for different *p* and $N_2 = 50$.

p	2	3	5	10	20
SIA	**0.163** ** (0.259)**	** 0.117 ** **(0.170)**	**0.099 ** ** (0.102)**	**0.086 ** ** (0.055)**	**0.245 ** **(0.215)**
AD-GD	0.122 (0.259)	0.112 (0.171)	0.068 (0.101)	0.052 (0.066)	0.208 (0.242)
EM	0.158 (0.258)	0.105 (0.173)	0.078 (0.095)	0.070 (0.054)	0.061 (0.061)
MClust	0.154 (0.322)	0.102 (0.148)	0.052 (0.088)	0.025 (0.028)	0.017 (0.015)

Significant values are in bold.

**Table 10:** ARI (mean and SD) for different *p* and $N_2 = 100$.

p	2	3	5	10	20
SIA	0.088 (0.073)	** 0.134 ** **(0.105)**	** 0.089** **(0.112)**	**0.108 ** **(0.077)**	0.474 (0.221)
AD-GD	0.066 (0.075)	0.125 (0.106)	0.081 (0.114)	0.094 (0.084)	**0.538** **(0.260)**
EM	0.084 (0.070)	0.108 (0.098)	0.073 (0.088)	0.089 (0.078)	0.078 (0.075)
MClust	**0.131 ** **(0.240)**	0.110 (0.087)	0.057 (0.079)	0.012 (0.014)	0.003 (0.006)

Significant values are in bold.

## Simulation studies

We simulate over 2000 datasets from mixtures with non-Gaussian components and with varying (i) cluster separation (ii) covariance structures and imbalance across component sizes and (iii–iv) number of components. In all these settings, we vary the dimensionality as well. Table 2 shows a summary of all the settings and the sections in which the experiments are discussed. Experiments to evaluate MPKL as a model selection criterion are in Appendix L.2.

We evaluate the clustering performance of misspecified GMM using four inference techniques—EM , AD-GD, MClust (that implements EM for GMMs with 14 different, including equi-covariance, constraints) and our SIA algorithm. Note that in SIA, EM and AD-GD, the same model (unconstrained GMM) is fitted; the difference lies only in the inference approach. MClust fits different models (constrained GMMs) altogether. We use it as a baseline method to compare the performance of the softer regularization-based approach in SIA with the harder constraint-based approach in MClust, two different ways of providing inductive bias to the models.

All the algorithms are initialized using K-Means and are run till convergence. In MClust, instead of the default initialization with model-based agglomerative clustering, we use K-Means for a fair comparison; the best among its 14 internal models are selected using BIC.

### Varying cluster separation

We simulate data from p-dimensional mixture model with 3 Gaussian components, ${\mathcal {N}}(0.5\lambda$ $(1,\dots ,1)_p,{\mathbb {I}}_p)$, ${\mathcal {N}}((0,\dots ,0)_p,{\mathbb {I}}_p)$, ${\mathcal {N}}(-0.5\lambda (1,\dots ,1)_p,{\mathbb {I}}_p)$, where $\lambda$ is a scaling factor which controls the cluster separation and ${\mathbb {I}}_2$ is a unit covariance matrix. We evaluate the performance for $\lambda$ values in $\{3,4,5,7\}$ and *p* values in $\{2,3,5,10,20\}$. For each value of *p*, we sample $100 \times p$ datapoints from each of the 3 components. These sampled datapoints are cubed so that none of the components is normally distributed. 50 datasets are simulated for each setting. The results for different values of *p* are given in Tables 3, 4, 5, 6 and 7. At higher values of *p*, SIA outperforms all other methods and at lower values of *p*, SIA outperforms EM and GD. When the dimensionality is small and the cluster separation is poor, we find that the performance of Mclust is slightly better than that of SIA.

### Varying covariance structures and unbalanced mixtures

We sample from a 2 component *p*-dimensional GMM whose means are $(-0.5,\dots ,-0.5)_p$ and $(0.5,\dots ,0.5)_p$ and covariances matrices are $\Sigma _k = (\Sigma _k^{1/2} (\Sigma _k^{1/2})^T )$. The parameters of $\Sigma _k^{1/2}$ are sampled randomly to capture different covariance structures. We demonstrate in Appendix N that this sampling approach indeed yields diverse covariance structures. The simulated datapoints are then cubed (for misspecification). Keeping the number of data points ($N_1 \times p$) in cluster 1 constant at $100 \times p$, we vary the number of datapoints ($N_2 \times p$) in cluster 2 as $\{ 20 \times p,50 \times p,100 \times p \}$. We simulate 50 different datasets for each setting. We vary the dimensionality *p* as $\{2,3,5,10,20 \}$. The results are given in Tables 8, 9 and 10. We observe that when the dimensionality is low, clustering performance is better at higher imbalance. Appendix K shows an illustration. At higher values of *p*, clustering performance is better at lower imbalance. Overall, we find that SIA performs on par or better than EM, AD-GD and MClust.

**Table 11:** Clustering performance (ARI and RI) on ten real datasets.

				ARI				RI			
Dataset	n	p	K	SIA	EM	AD-GD	MClust	SIA	EM	AD-GD	MClust
I	178	13	3	**0.63**	0.462	0.375	0.62	**0.835**	0.742	0.721	0.829
II	150	4	3	**0.92**	0.90	0.90	0.90	**0.965**	0.957	0.957	0.957
III	4177	8	3	**0.130**	0.121	0.089	0.072	**0.587**	0.582	0.577	0.566
IV	168	148	9	**0.11**	–	0	–	**0.613**	–	0	–
V	130	206	2	**0.023**	–	0	−0.01	**0.513**	-	0	0.496
VI	569	30	2	**0.610**	0.593	0.213	0.593	**0.805**	0.797	0.621	0.797
VII	200	5	4	**0.822**	0.818	0	0.322	**0.933**	0.931	0	0.744
VIII	400	6	6	**0.188**	0.116	0.112	0.132	**0.651**	0.626	0.599	0.548
IX	88	17	2	0.866	0.783	0.9101	**1.0**	0.933	0.891	0.955	**1.0**
X	17898	8	2	**0.402**	0.349	0.348	0.344	**0.770**	0.739	0.738	0.736

Significant values are in bold.

**Table 12:** Clustering results for wine dataset.

S	*K*	Algorithm	LL	AIC	KLF	KLB	MPKL	ARI
1	2	EM	−2983.6	6385.3	86.3	39.5	46.8	0.46
2	2	EM	−3117.4	6652.9	17.2	14.8	2.3	0.57
3	2	EM	−3083.0	6584.0	22.2	17.36	4.8	0.59
4	2	AD-GD	−3081.6	6581.2	56.1	29.6	26.5	0.55
5	2	SIA	−3072.0	6562.0	42.1	27.5	14.6	0.57
6	3	EM	−2901.0	6430.0	321.29	91.44	229.8	0.46
7	3	EM	−2915.7	6459.4	93.25	118	51.68	0.62
8	3	AD-GD	−3030.04	6688.8	165.9	152.1	50.7	0.38
9	3	SIA	−2921.45	6470.9	57.7	60.8	**14.66**	**0.63**
10	4	EM	−2637.7	**6113.4**	2.86e8	407.7	134e8	0.52
11	4	EM	−2660.7	6158.0	513e7	362.1	513e7	0.52
12	4	AD-GD	−2910.7	6659.4	1192.4	364.0	486.65	0.35
13	4	SIA	−2850.5	6539.0	519.0	304.9	214.05	**0.67**

Significant values are in bold.

**Table 13:** Results for the best model for each *K* (S3, S9, S13 in Table 12). $\textcircled {1}$, $\textcircled{2}$, $\textcircled{3}$: 3 wine types.

K	$\textcircled{1}$	$\textcircled{2}$	$\textcircled{3}$
2	59		40
		71	8
3	56	6	
		56	7
	3	9	41
4	35	3	
		63	0
	75	5	48
	17		

## Evaluation on real datasets

We evaluate SIA on real datasets: (I) Wine^45^, (II) IRIS^46^, (III) Abalone^47^), (IV) Urban Land cover^48^, (V) LUSC RNA-Seq^49^), (VI) Breast Cancer^50^, (VII) Crabs^51^, (VIII) Wholesale^52^, (IX) Ceramics^53^, and (X) HTRU^54^.

The datasets are diverse with respect to values of $n,p \text { and } K$ and such that their underlying clusters do not appear to be Gaussian or well-separated (as seen through their scatter-plots). EM, AD-GD, MClust and SIA are initialized using K-Means, with 10 different random initializations within K-Means, and the best result obtained is reported.

Table 11 shows the dataset statistics and the ARI obtained (using the provided ground truth) by each method.

In datasets IV and V, the number of observations is lesser than the dimensions ($n < p$), EM fails to run and AD-GD assigns all the points to a single cluster.

### Case study

The Wine dataset contains the results of a chemical analysis of wines, yielding 13 features, from 3 different cultivars. We use this dataset, without the labels of wine types, and fit EM, AD-GD, MClust and SIA for $K \in \{ 2,3,4 \}$. The log-likelihood (LL), MPKL, KLDivs, AIC and ARI values are shown in Table 12. For each *K* we show the results for multiple runs with different initializations.

For $K=2$, among the 3 EM solutions, while clustering S1 has better LL, its MPKL is higher (worse) than those obtained by the other clustering outputs (S2 and S3). So, even among EM solutions, by trading off likelihood for an improvement in MPKL, we can choose better clustering solutions with higher accuracy. The LL of SIA is better than that of AD-GD (which was used in step 1 of SIA) indicating that its penalty term can aid in escaping local maxima. The ARI from SIA (S5) is comparable to the best results from EM. We observe similar trends for $K=3$. Among EM solutions (S6 and S7), the solution with better MPKL (S7) achieves a better ARI. Again, the LL of SIA (S9) is better than that of AD-GD (S8), which was used in step 1 of SIA. The best ARI is achieved by SIA which also has the lowest MPKL value. For $K=4$, we observe that AD-GD solution (S12) does not have high LL, nor does it have a low MPKL value. When SIA step-2 is initialized with the output of EM (S13), we observe a decrease in MPKL and an improvement in ARI over both EM and AD-GD. The confusion matrices of models with the best MPKL values for each *K* is in Table 13. Misspecification of the components is evident in the data scatterplot and S9 visually has the most well separated clusters (both in Appendix M).

## Conclusion

In this paper, we illustrate and discuss the problem of inferior solutions that occur while clustering with misspecified GMMs. They differ in their characteristics from spurious solutions in terms of the asymmetry of component orientation and sizes, and frequency of occurrence. Our theoretical analysis highlights a new relation between such asymmetry of fitted components and misspecification. Further investigation of this connection would be interesting to explore in the future.

We propose a new penalty term based on the Kullback Leibler divergence between pairs of fitted components that, by design, avoids inferior solutions. We prove that the penalized likelihood is bounded and avoids degeneracy. Gradient computations for this penalized likelihood is difficult but Automatic Differentiation can be done effortlessly. We develop algorithms SIA for clustering and MPKL for model selection, and evaluate their performance, on clustering synthetic and real datasets with misspecified GMMs. The superiority of SIA for misspecification is primarily demonstrated through extensive experimentation. Our simulation study reveals that SIA works well in several cases of misspecification that we examine, particularly when the cluster separation is low. At high cluster separation, the effect of misspecification on clustering is less acute and other methods, like MClust, also perform well, and the performance of SIA is comparable. Although the penalty term in SIA is designed to penalize component asymmetry, SIA performs well even when there are significant differences with respect to orientations and sizes of the true components in our simulation study. This is because SIA does not use hard constraints on the orientations and sizes and the functional penalization approach enables a data-dependent discovery of the underlying clusters. Nevertheless, it is possible in some cases for SIA to fit symmetric components when the underlying clusters have covariances with different orientations and sizes. The use of our proposed MPKL criterion in conjunction with other likelihood-based criteria like AIC or BIC, as discussed in our case study, can guide the user to find good clustering solutions in such cases. Statistical guarantees with this penalized likelihood approach and extensions to high-dimensional settings require further work and would be of theoretical and practical interest.

### Supplementary Information

Supplementary Information.

## Data Availability

Python implementation of our algorithms and experiments available at https://bitbucket.org/cdal/sia/.
