# Supplementary material for: Avoiding inferior clusterings with misspecified Gaussian mixture models
Source: Sci Rep. 2023 Nov 6;13:19164. doi: 10.1038/s41598-023-44608-3 (PMC10628229; doi:10.1038/s41598-023-44608-3)
Supplement: Supplementary file 1 — Supplementary Information. [file 41598_2023_44608_MOESM1_ESM.pdf]

Appendix

A Symbols and Notation

Symbol	Meaning
f	overall probability density from which data is sampled
g	misspecified model for data sampled from f
f_i	probability density of the i-th component
π_i	mixing proportions or weight of the i-th component
θ_i	parameters of i-th mixture component
θ	all parameters of the model
θ^*	true parameters of the model from which data is sampled
Θ	set of all possible parameters i.e. the entire parameter space
$\mathbf{F}_i$	factors associated with i-th datapoint
K	total of number of components in the model
$k, k^{'}, k_1, k_2$	indices over the total number of components K
$\mathbf{X}_i$	i-th random datapoint
$\mathbf{x}_i$	i-th observed datapoint
$\mathbf{X}$	all the n random datapoints
$\mathbf{x}$	all the n observed datapoints
n	Total number of iid datapoints considered
p	Total number of dimensions or features of the data
p_e	Total number of free parameters in the model
$\mathcal{L}(\theta)$	Log-Likelihood of observing $\mathbf{x}$ if the parameters were θ
$\mathcal{M}(\theta)$	Penalized SIA Log-Likelihood if the parameters were θ
$\mathcal{N}$	density of multi-variate Gaussian distribution
$\mathcal{B}$	Bernoulli distribution
μ_i, μ_{ij}	mean of i-th component of a GMM and its value of along j-th dimension respectively
Σ_i, Σ_{ijk}	Covariance matrix of i-th component of a GMM and its jk-th element respectively
$\mathbf{U}_i$	square-root of i-th covariance matrix Σ_i
$\hat{a}^t$	Estimate of parameter a at the end of iteration t
c, C	Constants independent of model parameters
λ	factor controlling the cluster separation in simulations
λ_k	hyperparameter controlling the fitted component covariance
$\mathbb{I}$	Identity matrix
ϵ	learning rate in the vanilla gradient descent step
γ	convergence threshold for stopping criterion
b	factor controlling the variance of the contaminating component
$\tau(\bar{\theta})$	Asymmetry coefficient for inferred GMM parameters $\bar{\theta}$
C_2	constant dependent on the true model parameters
D, D'	Discrete measures
$\Pi(D, D')$	Set of all Couplings between D, D'
h	a coupling between D, D'
G^*	3-Component corrupted GMM
G	2-Component GMM being fit to G^*

Table 1. Symbols used in the paper

B Spurious Solutions

Spurious solutions are local maximizers of the likelihood function but lack real-life interpretability and hence do not provide a good clustering of the data. It is a consequence of the unboundedness of the GMM likelihood function for unrestricted component covariance matrices. As discussed in [1], spurious solutions may be obtained when:

- a fitted component has very small non-zero variance for univariate data or generalized covariance, i.e., the determinant of the covariance matrix, for multivariate data. Such a component corresponds to a cluster containing very few data points close together or, for multivariate data, in a lower dimensional subspace.
- the model fits a small localized random pattern in the data instead of an underlying group structure. Such solutions have very few points in one cluster with little variation, compared to other clusters, in the cluster's axes, or for multivariate data, small eigenvalues for the component covariance matrix.
- where the likelihood increases by fitting the covariance matrix of a component on just one or a few datapoints distant from the other samples.

In some cases, e.g., when a component is fitted over very few datapoints, spurious solutions lead to singularities in the component covariance matrices that can be detected during EM inference. There are other cases as well, when the parameters lie close to the boundary of the parameter space, when the component covariance matrices are not singular but may be close to singular for some components.

[1] observed that convergence to spurious solutions happen rarely; they are dependent on initialization – often occurring only for some initializations; and convergence to non-spurious global maxima becomes difficult with increasing dimensionality.

C Model Selection

Information Criterion-based model selection methods are designed to favor parsimony in modeling, by penalizing overfitting of the model[2]. Common likelihood-based criteria include Akaike Information Criterion (AIC):

$$\text{AIC} := 2p_e - 2\mathcal{L}(\hat{\theta}),$$

and Bayesian Information Criterion (BIC):

$$\text{BIC} := p_e \ln(n) - 2\mathcal{L}(\hat{\theta}),$$

where p_e is the number of parameters to be estimated in the model.

Traditional likelihood based model selection criteria such AIC and BIC cannot used reliably when there is misspecification as they assume that the model has been rightly specified [2, 3]. There exists more generalized information criteria such as Takeuchi Information Criteria [4] which can accomodate misspecified models; however their practical use is limited by the requirement to evaluate Hessians with respect to model parameters. It has been pointed out that MLE based criteria such as AIC and BIC are based on asymptotic normality and hence, cannot be used for model selection in high-dimensions where $n << p$ [5, 6, 7]. Moreover, in high dimensions, it has been observed that BIC tends to underestimate the number of components whereas AIC overestimates the number of components [8].

D Automatic Differentiation based Gradient Descent

The contents of this section are from our previous work [9], with examples from [10].

D.1 Gradient Descent and Automatic Differentiation

Automatic Differentiation (AD) (also known as algorithmic differentiation) is a suite of computational techniques for efficient and accurate evaluation of derivatives of numeric functions. In traditional GD based inference, gradients are required in closed form which becomes laborious or intractable to derive as the model complexity increases, e.g., for MFA. To automate the computation of derivatives three classes of techniques have been developed: (a) Finite Differentiation (FD) (b) Symbolic Differentiation (SD) and (c) Automatic or Algorithmic Differentiation (AD). Although easy to code, FD is slow at high dimensions and susceptible to floating point errors. SD provides exact symbolic expressions of derivatives but has high computational complexity, in both time and memory and cannot be used when functions are defined using programmatic constructs such as conditions and loops. The complexity and

Table 2. Iterations of the logistic map $l_{n+1} = 4l_n(1-l_n)$, $l_1 = x$ and the corresponding derivatives of l_n with respect to x, illustrating expression swell (from [10])

n	l_n	$\frac{d}{dx}l_n$	$\frac{d}{dx}l_n$ (Simplified form)
1	x	1	1
2	$4x(1-x)$	$4(1-x)-4x$	$4-8x$
3	$16x(1-x)(1-2x)^2$	$16(1-x)(1-2x)^2 - 16x(1-2x)^2 - 64x(1-x)(1-2x)$	$16(1-10x+24x^2-16x^3)$
4	$64x(1-x)(1-2x)^2\ (1-8x+8x^2)^2$	$128x(1-x)(-8+16x)(1-2x)^2(1-8x+8x^2)+64(1-x)(1-2x)^2(1-8x+8x^2)^2 - 64x(1-2x)^2(1-8x+8x^2)^2 - 256x(1-x)(1-2x)(1-8x+8x^2)^2$	$64(1-42x+504x^2-2640x^3+7040x^4-9984x^5+7168x^6-2048x^7)$

errors in both FD and SD increase for the computation of higher derivatives and partial derivatives of vector-valued functions. AD overcomes all these limitations of FD and SD. It provides efficient and accurate numerical evaluation of derivatives without requiring closed form expressions.

The numerical computation of a function can be decomposed into a finite set of elementary operations. These operations most commonly include arithmetic operations and transcendental function evaluations. The key idea of AD is to numerically compute the derivative of a function by combining the derivatives of the elementary operations through the systematic application of the chain rule of differential calculus. The efficiency of the computation is improved by storing evaluated values of intermediate sub-expressions that may be re-used. Backpropagation, used to train neural networks, is a specific form of AD which is more widely applicable. We refer the reader to recent surveys [10, 11] for more details.

Efficient implementations of AD are available in several programming languages and frameworks, e.g. Python [12], R [13], Pytorch [14] and Stan [15]. Many first and second order gradient-based optimization algorithms are implemented in these libraries. In this paper, we use Adam, a first order method that computes individual adaptive learning rates for different parameters from estimates of first and second moments of the gradients [16].

D.2 Illustration of Automatic Differentiation

In symbolic differentiation, we first evaluate the complete expression and then differentiate it using rules of differential calculus. First note that a naive computation may repeatedly evaluate the same expression multiple times, e.g., consider the rules:

$$\frac{d}{dx}\left(F(x)+G(x)\right) \rightsquigarrow \frac{d}{dx}F(x)+\frac{d}{dx}G(x) \tag{1}$$

$$\frac{d}{dx}\left(F(x)\,g(x)\right) \rightsquigarrow \left(\frac{d}{dx}F(x)\right)G(x)+F(x)\left(\frac{d}{dx}G(x)\right) \tag{2}$$

Let $H(x) = F(x)G(x)$. Note that $H(x)$ and $\frac{d}{dx}H(x)$ have in common: $F(x)$ and $G(x)$, and on the right hand side, $F(x)$ and $\frac{d}{dx}F(x)$ appear separately. In symbolic differentiation we plug the derivative of $F(x)$ and thus have nested duplications of any computation that appears in common between $F(x)$ and $\frac{d}{dx}F(x)$. In this manner symbolic differentiation can produce exponentially large symbolic expressions which take correspondingly long to evaluate. This problem is called **expression swell**. To illustrate the problem, consider the following iterations of the logistic map $l_{n+1} = 4l_n(1-l_n)$, $l_1 = x$ and the corresponding derivatives of l_n with respect to x. Table 2 clearly shows that the number of repetitive evaluations increase with n.

If the symbolic form is not required and only numerical evaluation of derivatives is required, computations can be simplified by storing the values of intermediate sub-expressions. Further efficiency gains in computation can be achieved by interleaving differentiation and simplification steps. The derivative of $l_{n+1} = 4l_n(1-l_n)$ can be found using the chain rule $\frac{dl_{n+1}}{dl_n}\frac{dl_n}{dl_{n-1}}\ldots\frac{dl_1}{dl_x}$ which simplifies to $4(1-l_n-l_n)4(1-l_{n-1}-l_{n-1})\ldots4(1-x-x)$. Note that evaluation in AD is computationally linear in n (because we add only one $(1-l_n-l_n)$ for each increase by 1). This

linear time complexity is achieved due to ‘carry-over’ of the derivatives at each step, rather than evaluating the derivative at the end and substituting the value of x.

The Python code below shows the simplicity of the implementation for this problem.

```
from autograd import grad
def my_func(x,n):
    p = x
    y = x * (1 - x)
    for i in range(n):
        y = y*(1 - y)

    return y
grad_func = grad(my_func)
grad_func(0.5,4)
```

Consider the following recursive expressions: $l_0 = \frac{1}{1+e^x}$, $l_1 = \frac{1}{1+e^{l_0}}$, ..., $l_n = \frac{1}{1+e^{l_{n-1}}}$ We evaluate the derivative of l_n with respect to x and compare the runtime in Mathematica (SD) vs PyTorch (AD) for various values of n. As n increases, it is expected that runtime also increases. It can be seen from the results in Table 3 that runtime increases linearly for AD (using PyTorch) whereas it increases exponentially for SD (using Mathematica).

Table 3. Average runtime (over 1000 runs)

n	AD	SD
1	0.00013	0.00000
5	0.00030	0.00005
10	0.00051	0.00023
50	0.00293	0.00437
100	0.00433	0.15625
200	0.00917	1.45364

D.3 Reparametrizations for AD-GD inference on GMMs

As mentioned in section 2, EM solves three main problems in GMM inference. In AD-GD, *Problem 1* is solved inherently because we do not need to express the gradients in closed form by virtue of using AD. Further, the second-order Hessian matrix may also be evaluated using AD, enabling us to use methods with faster convergence. To tackle *Problem 2*, instead of gradients with respect to Σ_k, we compute the gradients with respect to $\mathbf{U}_k$, where $\Sigma_k = \mathbf{U}_k \mathbf{U}_k^T$. We first initialize $\mathbf{U}_k$ as identity matrices. Thereafter, we keep adding the gradients to the previous estimates of $\hat{\mathbf{U}}_k^{t+1}$, i.e.

$$\hat{\mathbf{U}}_k^{t+1} := \hat{\mathbf{U}}_k^t + \epsilon \frac{\partial \mathcal{L}}{\partial \mathbf{U}_k};\ \hat{\Sigma}_k^{t+1} := \hat{\mathbf{U}}_k^{t+1} \hat{\mathbf{U}}_k^{t+1^T} \quad (3)$$

where ϵ is the learning rate and superscripts $t, t+1$ denote iterations in GD. If the gradients are evaluated with respect to Σ_k directly, there is no guarantee that updated $\hat{\Sigma}_k^{t+1} = \hat{\Sigma}_k^t + \epsilon \frac{\partial \mathcal{L}}{\partial \Sigma_k}$ will still remain PD. However, if the gradients are evaluated with respect to $\mathbf{U}_k$, by construction $\hat{\Sigma}_k^{t+1}$ will always remains PD. Cholesky decomposition for reparameterizing Σ_k can also be used [17]. *Problem 3* is solved by using the log-sum-exp trick [18]. We start with unbounded α_k as the log-proportions: $\log \pi_k = \alpha_k - \log(\sum_{k'=1}^K e^{\alpha_{k'}})$. We need not impose any constraints on α_k as the final computation of π_k automatically leads to normalization, because $\pi_k = \frac{e^{\alpha_k}}{\sum_{k'=1}^K e^{\alpha_{k'}}}$. Therefore, we reparametrize a constrained optimization problem into an unconstrained one (without using Lagrange multipliers) and we can update $\hat{\pi}_k$ as follows:

$$\hat{\alpha}_k^{t+1} := \hat{\alpha}_k^t + \epsilon \frac{\partial \mathcal{L}}{\partial \alpha_k};\ \hat{\pi}_k^{t+1} := \frac{e^{\hat{\alpha}_k^{t+1}}}{\sum_{k'=1}^K e^{\hat{\alpha}_{k'}^{t+1}}} \quad (4)$$

E Performance of EM and AD-GD on Misspecified GMM: Empirical Results

E.1 EM and AD-GD: A comparison of clustering performance

We compare the clustering performance of AD-GD and EM on pure GMM data as well as non-GMM (misspecified) data. We do so by simulating 3600 datasets as described below and running both the algorithms with matching

settings with respect to random initialization, number of iterations (maximum of 100 iterations) and convergence threshold (1e-5). The details of the simulations and results are given below.

Pure GMM data : We simulate datasets from 36 different 2–dimensional 3–component Gaussian Mixture Models that differ in their means and covariance matrices. This is to test different types of data - from well-separated components to highly overlapping components. The GMM parameters are varied as follows:

- First, we construct a 2×1 vector $\mathbf{v}_k$ and a 2×2 matrix $\mathbf{Z}_k$ for each of these 3 components, where each element of the vectors and matrices are sampled from a standard normal distribution.
- The mean vector of the kth component is obtained by multiplying $\mathbf{v}_k$ by a factor k_μ i.e. $\mu_k = k_\mu \mathbf{v}_k$. This factor k_μ is varied from 0.25 to 1.5 in steps of 0.25, i.e., the factor k_μ takes one of the six values in {0.25, 0.5, 0.75, 1, 1.25, 1.5}. Higher the value of k_μ, more the separation between the means of components.
- The $\mathbf{Z}_k$ vector is added to the 2×2 Identity matrix $\mathbb{I}$ (This step ensures the PD of the covariance matrix that is computed in the later steps).
- We multiply the matrix $\mathbf{Z}_k + \mathbb{I}$ by k_Σ to obtain $\mathbf{U}_k$. The scaling factor k_Σ is varied from 0.05 to 0.65 in steps of 0.1, i.e., the scaling factor takes one of the six values in {0.05, 0.15, 0.25, 0.35, 0.45, 0.55}. The covariance matrix of the kth component is $\Sigma_k = \mathbf{U}_k \mathbf{U}_k^T$. Higher the scaling factor k_Σ, more the inter-component overlap.

By choosing six different values of k_μ and k_Σ each, we simulate 36 (6x6) GMM parameters with varying cluster separation. For each these 36 GMM parameters, we simulate 50 different datasets each containing 300 samples, for a total of 1800 datasets. Both the methods (EM and AD-GD) are run on each dataset with 50 random initializations and average ARI is computed for each combination of (k_μ, k_Σ).

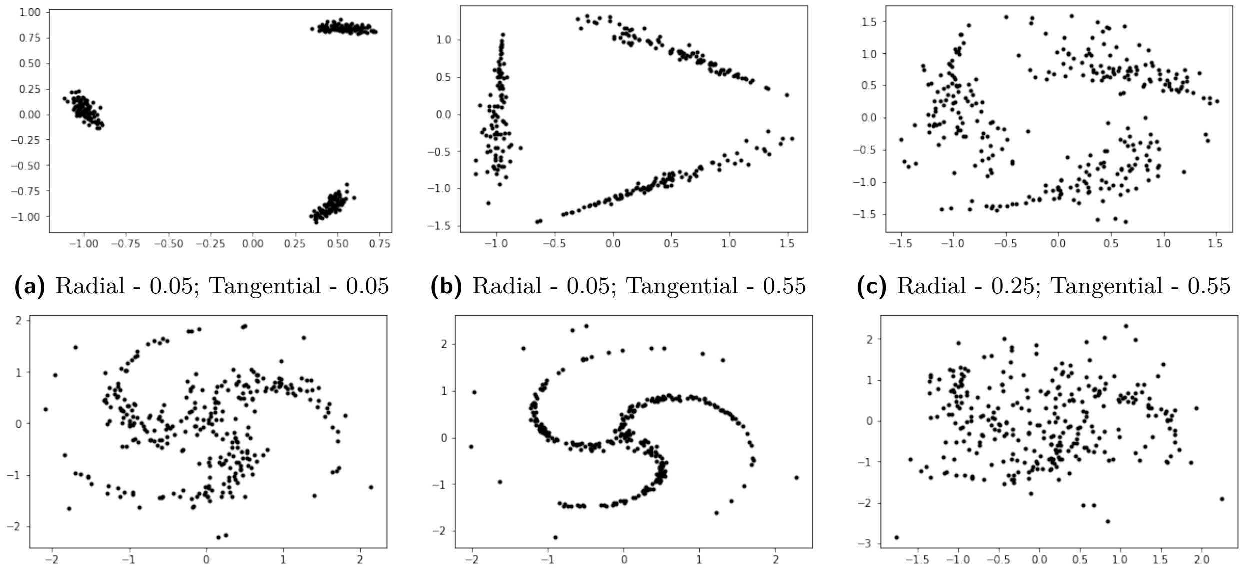

(a) Radial - 0.05; Tangential - 0.05 **(b)** Radial - 0.05; Tangential - 0.55 **(c)** Radial - 0.25; Tangential - 0.55

(d) Radial - 0.55; Tangential - 0.25 **(e)** Radial - 0.55; Tangential - 0.05 **(f)** Radial - 0.55; Tangential - 0.55

Figure 1. Data with completely different shapes can be simulated by varying the tangential and radial parameters of pinwheel dataset.

Misspecified datasets: The pinwheel data is generated by sampling from Gaussian distributions and then stretching and rotating the data in a controlled manner. The centers are equidistant around the unit circle. The variance is controlled by two parameters r and t, the radial standard deviation and the tangential standard deviation respectively. The warping is controlled by a third parameter, s, the rate parameter. A datapoint (x, y) belonging to component or arm k is generated as follows:

$$(x', y') \sim \mathcal{N}(0, \mathbb{I}) \tag{5}$$

$$(x, y) = ((rx'+1)\cos\theta_k + ty'\sin\theta_k, -(rx'+1)\sin\theta_k + ty'\cos\theta_k), \tag{6}$$

$$\text{where} \quad \theta_k = k\frac{2\pi}{K} + s \times e^{rx'+1}$$

The MATLAB [1] and Python[2] codes for generating pinwheel datasets are available online. We simulated 36 different pinwheel configurations by varying the radial and tangential components for 3-component mixture models using the Autograd package in Python. The rate parameter s of warping is fixed at 0.4 for all the 36 different combinations. This is to test multiple combinations – clusters with heavy tails to warped mixtures as shown in figure 1. The parameters of pinwheel datasets are chosen as follows:

- The radial parameter r is chosen to be one of the six values $\{0.05, 0.15, 0.25, 0.35, 0.45, 0.55\}$
- The tangent parameter t is chosen to be one of the six values $\{0.05, 0.15, 0.25, 0.35, 0.45, 0.55\}$

By choosing different values of r and t, we simulate 36 (6x6) set of pinwheel parameters. For each these 36 pinwheel parameters, we simulate 50 different datasets each with 300 samples, for a total of 1800 datasets. Please refer to figure 1 to see the various kinds of component shapes and separations that have been generated.

The tables below show the difference in average ARI between clusterings obtained by EM and those from AD-GD on GMM (table 4) and Pinwheel (table 5) datasets. The remaining tables (Tables 6 – 13) show the mean and standard deviations over the GMM and Pinwheel datasets for both EM and AD-GD. From the results in tables 4 and 5, we observe that in both the cases (misspecification and nomisspecification), EM inference outperforms AG-GD inference. [19]'s work on exact Newton's method using analytical derivatives also report similar results for non misspecified cases.

Table 4. Difference in average ARI of GMM datasets

$k_\mu \mid k_\Sigma$	0.05	0.15	0.25	0.35	0.45	0.55
0.25	0.56	0.52	0.44	0.40	0.37	0.36
0.5	0.43	0.55	0.49	0.44	0.41	0.40
0.75	0.36	0.51	0.52	0.48	0.46	0.42
1	0.36	0.48	0.55	0.53	0.50	0.50
1.25	0.30	0.47	0.55	0.58	0.54	0.54
1.5	0.33	0.51	0.58	0.60	0.61	0.56

Table 5. Difference in average ARI of Pinwheel datasets

$r \mid t$	0.05	0.15	0.25	0.35	0.45	0.55
0.05	0.18	0.30	0.41	0.50	0.51	0.08
0.15	0.47	0.50	0.56	0.58	0.57	0.24
0.25	0.51	0.58	0.61	0.62	0.61	0.47
0.35	0.30	0.39	0.45	0.53	0.49	0.43
0.45	0.12	0.17	0.20	0.24	0.27	0.30
0.55	0.05	0.08	0.12	0.16	0.18	0.19

Table 6. Average ARI of GMM datasets using EM

$k_\mu \mid k_\Sigma$	0.05	0.15	0.25	0.35	0.45	0.55
0.25	0.78	0.63	0.52	0.46	0.42	0.41
0.5	0.81	0.79	0.68	0.59	0.53	0.51
0.75	0.84	0.84	0.78	0.70	0.64	0.57
1	0.84	0.85	0.83	0.77	0.70	0.67
1.25	0.84	0.86	0.84	0.82	0.75	0.71
1.5	0.83	0.86	0.86	0.83	0.81	0.74

Table 7. Average ARI of GMM datasets using AD-GD

$k_\mu \mid k_\Sigma$	0.05	0.15	0.25	0.35	0.45	0.55
0.25	0.23	0.11	0.08	0.06	0.06	0.05
0.5	0.38	0.24	0.18	0.15	0.12	0.11
0.75	0.47	0.33	0.26	0.21	0.18	0.16
1	0.48	0.37	0.28	0.24	0.20	0.17
1.25	0.54	0.39	0.29	0.24	0.21	0.17
1.5	0.50	0.35	0.28	0.23	0.20	0.18

[1]https://github.com/duvenaud/warped-mixtures/blob/master/data/pinwheel.m
[2]https://github.com/HIPS/autograd/blob/master/examples/data.py

Table 8. Average ARI of Pinwheel datasets using EM

$r \mid t$	0.05	0.15	0.25	0.35	0.45	0.55
0.05	0.95	1.00	1.00	1.00	0.95	0.48
0.15	0.95	0.97	1.00	0.98	0.93	0.58
0.25	0.88	0.95	0.95	0.94	0.91	0.74
0.35	0.60	0.69	0.73	0.78	0.72	0.64
0.45	0.36	0.41	0.41	0.44	0.45	0.46
0.55	0.23	0.26	0.28	0.31	0.32	0.32

Table 9. Average ARI of Pinwheel datasets using AD-GD

$r \mid t$	0.05	0.15	0.25	0.35	0.45	0.55
0.05	0.77	0.70	0.59	0.50	0.44	0.40
0.15	0.48	0.47	0.44	0.40	0.36	0.34
0.25	0.37	0.37	0.34	0.32	0.29	0.27
0.35	0.30	0.30	0.28	0.25	0.23	0.21
0.45	0.24	0.24	0.22	0.20	0.18	0.16
0.55	0.19	0.18	0.17	0.15	0.14	0.12

Table 10. Std. Deviation of ARI of GMM datasets using EM

$k_\mu \mid k_\Sigma$	0.05	0.15	0.25	0.35	0.45	0.55
0.25	0.30	0.27	0.25	0.24	0.22	0.23
0.5	0.29	0.25	0.26	0.27	0.26	0.25
0.75	0.26	0.23	0.25	0.26	0.26	0.27
1	0.25	0.23	0.22	0.26	0.26	0.26
1.25	0.25	0.24	0.23	0.22	0.26	0.26
1.5	0.24	0.23	0.22	0.23	0.22	0.26

Table 11. Std. Deviation of ARI of GMM datasets using AD-GD

$k_\mu \mid k_\Sigma$	0.05	0.15	0.25	0.35	0.45	0.55
0.25	0.21	0.13	0.09	0.08	0.07	0.05
0.5	0.26	0.21	0.17	0.15	0.12	0.10
0.75	0.25	0.23	0.21	0.19	0.16	0.14
1	0.28	0.23	0.21	0.19	0.17	0.15
1.25	0.29	0.24	0.21	0.18	0.16	0.15
1.5	0.27	0.25	0.23	0.20	0.18	0.16

Table 12. Std. Deviation of ARI of Pinwheel datasets using EM

$r \mid t$	0.05	0.15	0.25	0.35	0.45	0.55
0.05	0.15	0.00	0.00	0.00	0.16	0.32
0.15	0.15	0.12	0.00	0.08	0.15	0.34
0.25	0.12	0.10	0.10	0.08	0.06	0.25
0.35	0.15	0.16	0.17	0.13	0.18	0.17
0.45	0.13	0.12	0.16	0.19	0.18	0.16
0.55	0.08	0.08	0.09	0.11	0.12	0.11

Table 13. Std. Deviation of ARI of Pinwheel datasets using AD-GD

$r \mid t$	0.05	0.15	0.25	0.35	0.45	0.55
0.05	0.03	0.04	0.04	0.04	0.05	0.05
0.15	0.04	0.04	0.05	0.04	0.05	0.04
0.25	0.04	0.04	0.04	0.04	0.04	0.04
0.35	0.03	0.03	0.04	0.04	0.03	0.03
0.45	0.03	0.03	0.03	0.03	0.03	0.03
0.55	0.03	0.03	0.03	0.03	0.03	0.03

E.2 Summary statistics of solutions in sets 1–4 for EM

Table 14. Summary statistics of EM clustering solutions over 100 random initializations on the Pinwheel dataset (shown in fig. 1), grouped into 4 sets based on AIC ranges. Mean and standard deviation of AIC, ARI, component weights and covariance determinants, over solutions in each set.

Set	AIC Range	AIC	ARI	π_1	π_2	π_3	$\lvert\Sigma_1\rvert$	$\lvert\Sigma_2\rvert$	$\lvert\Sigma_3\rvert$
1	771-773	771.9	0.625	0.257	0.265	0.477	0.0002	0.0005	0.125
		(0.0005)	(3e-16)	(5e-4)	(7e-5)	(1e-4)	(4e-7)	(1e-7)	(1e-4)
2	781-782	781.1	0.912	0.306	0.341	0.352	7e-4	0.01	0.01
		(2e-6)	(0)	(4e-6)	(3e-6)	(7e-6)	(1e-7)	(4e-7)	(2e-6)
3	786-787	786.8	0.652	0.257	0.266	0.475	2e-4	5e-4	0.157
		(0.001)	(0.002)	(5e-5)	(4e-4)	(4e-4)	2e-7	(6e-6)	(5e-4)
4	788-850	810.7	0.840	0.287	0.319	0.393	3e-3	0.04	0.84
		(10.84)	(0.084)	(0.022)	(0.009)	(0.03)	(1e-3)	(0.02)	(0.084)

F Additional Motivating Examples of Inferior Clusterings

F.1 Contamination with Student's T Distribution

As another motivating example, we illustrate the case where misspecification arises due to contamination by alternative distribution [20]. Consider a 2-dimensional GMM with 4 components, each with a unit spherical covariance. The means of the four components are set as given in Table 15 with $\beta = 3$. From each Gaussian

Table 15. Component (C) Means

	C-1	C-2	C-3	C-4
μ_1	-β	0	0	β
μ_2	0	β	-β	0

component, we sample 50 datapoints. Next, we contaminate the sampled points using a 2-dimensional 4-component multivariate t distribution with same means and covariance structures. From each multivariate t component, we sample an additional 50 datapoints. Thus each component has 100 samples and a dataset has 400 samples. We thus have samples from a Gaussian mixture contaminated by a Student t mixture, where the degree of contamination can varied by the Degrees of Freedom (D.o.F) of the multivariate t distribution. When the degree of freedom is high, the contamination is negligible. When the degree of freedom is low, the components based on t-distribution will have heavier tails, and thus the contamination is higher. By varying the degrees of freedom we can control the contamination and hence the misspecification.

We fit a 4-component GMM using EM (we obtain similar results using AD-GD as well). As seen from Fig. 2, the components tend to overlap with increasing misspecification (decreasing D.o.F). We observe that when the misspecification is high, some of the fitted components tend to have higher covariance compared to other components. We repeat the same experiment of corrupting the GMM with a multivariate t-distribution but with increasing the cluster separation by setting $\beta = 5$. The results are shown in Fig. 3 As seen, increasing cluster separation mitigates the affect of misspecification. A similar experiment on contamination with random noise is shown in Appendix F.2.

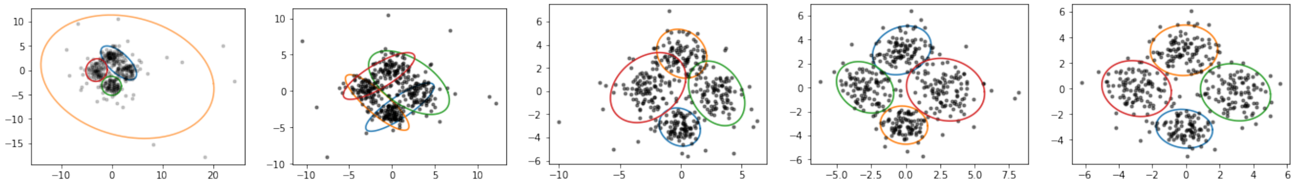

(a) D.o.F = 2 **(b)** D.o.F = 3 **(c)** D.o.F = 4 **(d)** D.o.F = 5 **(e)** D.o.F = 10

Figure 2. Illustration of GMM contaminated by Student t mixture with varying degree of freedom (D.o.F) at low cluster separation. Lower the D.o.F., higher the misspecification, and greater is the difference in orientation and size of the components fitted.

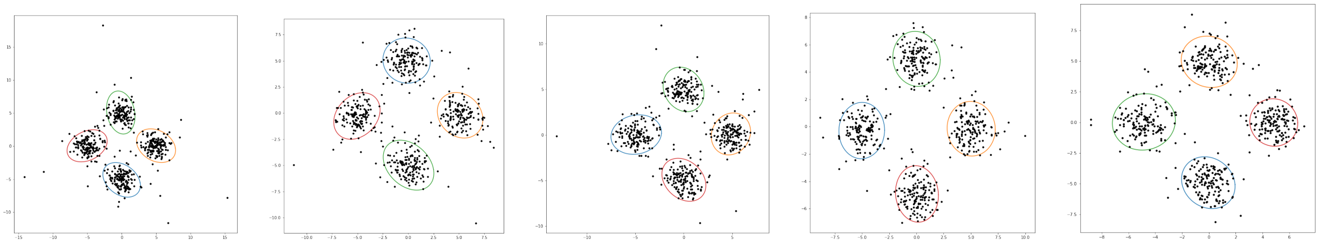

(a) D.o.F = 2 **(b)** D.o.F = 3 **(c)** D.o.F = 4 **(d)** D.o.F = 5 **(e)** D.o.F = 10

Figure 3. Illustration of GMM contaminated by Student t mixture with varying degree of freedom (D.o.F) at high cluster separation. Misspecification does not seem to affect the fitted components much, only the orientation of the fitted components is affected.

F.2 Contamination using uniform noise

We construct a four component GMM with unit covariances and means $\{(-3,0),(0,3),(0,-3),(3,0)\}$. We sample 50 datapoints from each component. We add datapoints sampled uniformly from the square $(-6,6)\times(-6,6)$ as noise. We fit a 4-component GMM using EM in each case. Fig. 5 shows that as we increase the number of noisy datapoints

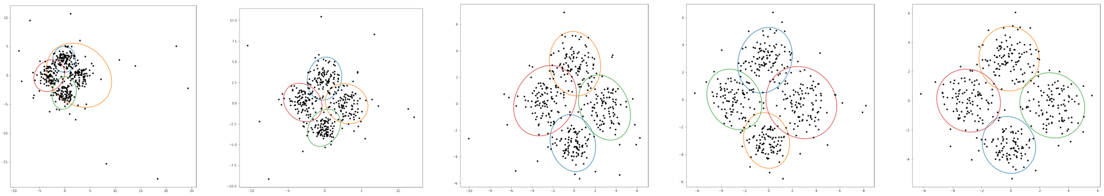

(a) D.o.F = 2 **(b)** D.o.F = 3 **(c)** D.o.F = 4 **(d)** D.o.F = 5 **(e)** D.o.F = 10

Figure 4. SIA improves the clustering performance when the Gaussian components are corrupted by a multivariate t-distribution - Refer to table 16

Table 16. Improvement in clustering performance before and after SIA step-2 for the contaminated case

	Before SIA Step 2					After SIA Step 2				
DoF	LL	KLF	KLB	MPKL	ARI	LL	KLF	KLB	MPKL	ARI
2	-1837.6	158.4	60.7	58.6	0.57	-1943.9	36.8	41.6	8.8	0.76
3	-1813.4	57.2	80.9	16.6	0.37	-1871.0	48.2	46.2	4.3	0.79
4	-1725.8	48.6	89.1	11.7	0.75	-1735.1	39.6	54.3	3.8	0.78
5	-1683.9	67.1	82.9	11.12	0.79	-1694.5	46.1	54.6	4.9	0.82
10	-1640.7	68.0	96.9	13.9	0.87	-1648.3	56.3	65.8	4.2	0.87

added to the our original data sampled from GMM, the adverse effect of misspecification on EM increases. Fig. 7 shows that SIA performs well and obtains good clustering. Table 17 shows the loglikelihood, KLF, KLB, MPKL and ARI values for each case, before and after step 2 of SIA. We repeat the same experiment of adding noisy data samples, but with increased cluster separation by choosing the means as $\{(-5,0),(0,5),(0,-5),(5,0)\}$. Fig. 6 shows that increasing the cluster separation mitigates the effect of misspecification with EM.

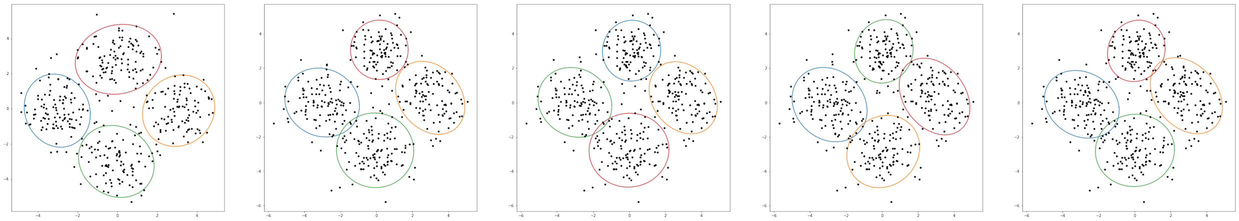

(a) # of noise samples = 10 **(b)** # of noise samples = 20 **(c)** # of noise samples = 30 **(d)** # of noise samples = 40 **(e)** # of noise samples = 50

Figure 5. As the number of noisy samples increases, the affect of misspecification also increases

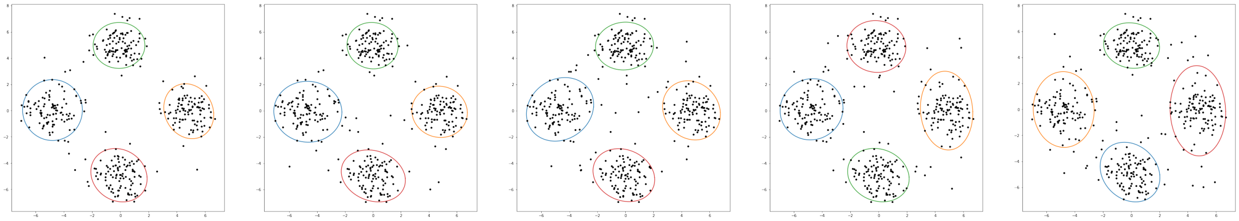

(a) # of noise samples = 10 **(b)** # of noise samples = 20 **(c)** # of noise samples = 30 **(d)** # of noise samples = 40 **(e)** # of noise samples = 50

Figure 6. Increasing the cluster separation mitigates the effect of uniform noise on specification

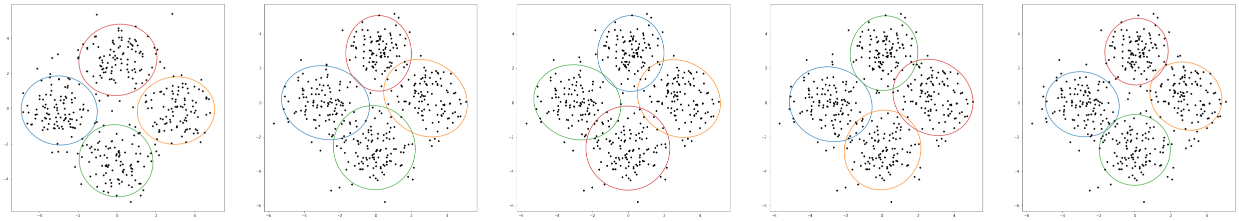

(a) # of noise samples = 10 **(b)** # of noise samples = 20 **(c)** # of noise samples = 30 **(d)** # of noise samples = 40 **(e)** # of noise samples = 50

Figure 7. SIA improves the clustering performance in the uniform noise case

Table 17. Improvement in clustering performance before and after SIA step-2 with increasing noisy samples $(10, \ldots, 50)$

	Before SIA Step 2					After SIA Step 2				
# of samples	LL	KLF	KLB	MPKL	ARI	LL	KLF	KLB	MPKL	ARI
10	-1676.9	75.6	85.2	4.4	0.91	-1681.3	69.0	70.6	1.2	0.92
20	-1719.6	85.2	68.2	8.4	0.87	-1737.8	51.3	45.0	2.4	0.89
30	-1761.1	64.0	87.2	6.9	0.88	-1778.4	44.2	52.8	2.3	0.89
40	-1806.2	78.1	67.3	5.6	0.87	-1815.9	55.2	51.7	2.2	0.89
50	-1839.6	79.4	67.1	6.2	0.87	-1845.5	70.2	65.7	2.0	0.88

F.3 Inferior Clusterings at High Dimensions: Corruption with a Student's t distribution

In this section, we show that under misspecification using Student's-t corruption, the asymmetric fitted components observed in 2-dimensional settings are also present for higher dimensions. Our simulation setting is as follows: There are 3 clusters whose means are given by $(0, \ldots, 0)_p; (-3, \ldots, -3)_p; (3, \ldots, 3)_p$ and all of them have unit covariances. For each cluster, $50 \times p$ datapoints are simulated from a Gaussian distribution and $50 \times p$ datapoints are simulated from a Student's-t distribution. The degrees of freedom of the Student's-t distribution is set to 2 or 10 depending on the degree of misspecification. We fit a 3 component GMM in this misspecified setting. Since it is not possible to visualize in high dimensional settings, to capture the asymmetry in the fitted components, we compute the maximum difference between the weights of the fitted components Δ_w, maximum difference between the determinants of fitted covariances Δ_c, and the ARI of the clustering solution. When the misspecification is small (D.o.F = 10), then the values of Δ_c and Δ_w should be relatively small compared to when the misspecification is large (D.o.F = 2). The dimensionality p to chosen from $\{2, 5, 10, 50, 100\}$. For each setting, we simulate 50 different datasets and the average results are given in table 18. We can see that increasing dimensionality (while keeping the proportion of corrupted points the same) increases the asymmetry of fitted components and the clustering performance deteriorates.

Table 18. Inferior Clusterings at High Dimensions: Corruption with a Student's t distribution

	D.o.F = 2			D.o.F = 10		
p	Δ_c	Δ_w	ARI	Δ_c	Δ_w	ARI
2	4e4	0.73	0.08	4e0	0.114	0.741
5	6e11	0.84	1e-4	8e2	0.497	0.271
10	8e21	0.83	0.0	2e5	0.569	0.024
50	4e101	0.78	0.0	8e24	0.656	0.0
100	1e189	0.77	0.0	1e47	0.658	0.0

F.4 Inferior Clusterings at High Dimensions: Contamination with uniform noise

In this section, we show that under misspecification using uniform noise, the asymmetric fitted components observed in 2-dimensional settings are also present for higher dimensions. Our simulation setting is as follows: There are 3 clusters whose means are given by $(0, \ldots, 0)_p; (-3, \ldots, -3)_p; (3, \ldots, 3)_p$ and all of them have unit covariances. For each cluster, $100 \times p$ datapoints are simulated from a Gaussian distribution. We add $u_n \times p$ datapoints sampled uniformly from the square $(-6, \ldots, -6)_p \times (6, \ldots, 6)_p$ as noise. The value of u_n is set to 10 or 50 to control for the degree of

misspecification. We fit a 3 component GMM in this misspecified setting. Since it is not possible to visualize in high dimensional settings, to capture the asymmetry in the fitted components, we compute the maximum difference between the weights of fitted components Δ_w, maximum difference between determinants of fitted covariances Δ_c, and the ARI of the clustering solution. When the misspecification is small ($u_n = 10$), then the values of Δ_c and Δ_w should be relatively small compared to when the misspecification is large ($u_n = 50$). The dimensionality p to chosen from $\{2, 5, 10, 50, 100\}$. For each setting, we simulate 50 different datasets and the average results are given in table 19. We can see that increasing dimensionality (while keeping the proportion of corrupted points the same) decreases the asymmetry in fitted components' weights and the clustering performance increases.

Table 19. Inferior Clusterings at High Dimensions: Contamination with uniform noise

	$u_n = 10$			$u_n = 50$		
p	Δ_c	Δ_w	ARI	Δ_c	Δ_w	ARI
2	0.56	0.015	0.93	2.88	0.038	0.92
5	0.98	0.004	0.99	17.65	0.025	0.99
10	1.15	0.002	0.99	42.21	0.014	0.99
50	1.42	0.00	1	104.80	0.003	1
100	1.19	0.00	1	101.25	0.001	1

G Theoretical Results

G.1 Proof of Theorem 1

G.1.1 Upper bound on the KL-divergences between the true distribution and fitted distribution

Let true distribution be $G^* = \pi\mathcal{N}(-\mu,\sigma^2)+\pi\mathcal{N}(\mu,\sigma^2)+(1-2\pi)\mathcal{N}(\mu,b^2\sigma^2)$. WLOG, assume that $\mu > 0$. Let the fitted distribution be $G'(\theta) = (1-\pi')\mathcal{N}(\mu_1,\sigma_1^2)+\pi'\mathcal{N}(\mu_2,\sigma_2^2)$, where $\theta = (\mu_1,\sigma_1^2,\mu_2,\sigma_2^2,\pi')$.

Let $\theta^* = (\mu,\sigma^2,\mu,\sigma^2,\pi)$. Clearly, $G'(\theta^*) \neq G^*$. [21] derive the upper bound on the KL-divergence between true distribution and fitted (misspecified) distribution, when there is only one unknown parameter; we follow a similar approach.

We give a brief definition of Couplings of discrete measures. We define two discrete measures $D = \sum_k \pi_k \delta_{\eta_k}$ and $D' = \sum_{k'} \pi'_{k'} \delta_{\eta_{k'}}$, where δ_η is the Dirac Delta function at $\eta = (\mu,\sigma^2) \in \mathbb{R}\times\mathbb{R}^+$. The set of Couplings $\Pi(D,D')$ between two measures D and D' is defined as

$$\Pi\left(D,D'\right) = \left\{T \in \mathbb{R}_+^{k\times k'} : T 1_{k'} = \pi, T^\top 1_k = \pi'\right\} \tag{7}$$

where $\pi = (\pi_1,\ldots,\pi_k)^T, \pi' = \left(\pi'_1,\ldots,\pi'_{k'}\right)^T$, and 1_k denotes a k -dimensional vector with all entries equal to 1. In other words, $\Pi(G,G')$ is the set of all joint distributions T on the space $[k]\times[k']$ such that the marginals of the distribution T are equal to π and π' [21]. Couplings can be viewed as a discretized version of Copulae [22, 23].

Lemma 1 of [24] gives an upper bound on the KL-divergence of a two mixtures in terms of their individual components as follows:

$$\mathrm{KL}\left(G^*,G'(\theta)\right) \leq \inf_{\mathbf{h}\in\Pi(D,D')} \sum_{ij} h_{ij}\mathrm{KL}\left(f_i,f'_j(\theta)\right) \tag{8}$$

where $\mathbf{h}$ is a coupling, and f_i and $f_j^{'}$ are individual components of the mixtures G and G' respectively.

Define

$$\bar{G} := G'(\bar{\theta}) \quad \text{where} \quad \bar{\theta} \in \arg\min_{\theta\in\Theta} \mathrm{KL}\left(G_*,G'(\theta)\right) \tag{9}$$

Therefore, from equation 8, we have

$$\mathrm{KL}\left(G^*,G'(\bar{\theta})\right) = \min_\theta \mathrm{KL}\left(G^*,G'(\theta)\right) \leq \min_\theta \inf_{\mathbf{h}\in\Pi(D,D')} \sum_{ij} h_{ij}\mathrm{KL}\left(f_i,f'_j(\theta)\right) \leq \inf_{\mathbf{h}\in\Pi(D,D')} \sum_{ij} h_{ij}\mathrm{KL}\left(f_i,f'_j(\theta^*)\right) \tag{10}$$

Let $\mathbf{h} = \begin{bmatrix} \pi & 0 \\ \pi & 0 \\ 1-\bar{\pi}-2\pi & \bar{\pi} \end{bmatrix}$

Substituting this in the RHS of the inequality 8 and setting $\theta = \theta^*$, we get

$$\mathrm{KL}\left(G^*,G'(\bar{\theta})\right) \leq (1-2\bar{\pi})(-\log b + 0.5b^2 - 0.5) + \bar{\pi}\frac{2\mu^2}{\sigma^2} =: C_2 \tag{11}$$

where, C_2 is a known constant that depends only on the parameters of the true distribution.

G.1.2 Lower bound on the KL-divergence between the true distribution and fitted distribution

One approach to lower bound the KL-divergence is to use the proof ideas from Pinsker's inequality [25, 26]. In particular, we use the data processing inequality to derive a lower bound on $\mathrm{KL}\left(G^*,G'(\bar{\theta})\right)$. Before we proceed with the proof, we need the following propositions.

Proposition 1 (Pinsker's inequality for Bernoulli Random Variables)**.** *Let $p,q \in [0,1]$ be the parameters of two Bernoulli distributions $\mathbb{P}$ and $\mathbb{Q}$. Then*

$$KL(\mathbb{P},\mathbb{Q}) \geq 2(p-q)^2. \tag{12}$$

Proposition 2 (The BH Bound)**.** *Let m,m' be univariate distributions with support on the real line $\mathbb{R}$.*

$$\mathrm{TV}(m,m') \leq \sqrt{1-e^{-KL(m,m')}}. \tag{13}$$

The proof of Proposition 1 can be found in [26]. Proposition 2 is a more stricter alternative to Pinsker's inequality using the Bertagonolle Huber bound [27] - refer to Clement Canonne's article for a more detailed review [3].

Consider the sets $\mathbb{R}^-, \mathbb{R}^+$ (i.e. the -ve and +ve real lines) in $\mathbb{R}$. Let the random variable X be distributed according to G^* and the random variable Y be distributed according to $G'(\bar{\theta})$. Consider the random variables $X_b = \mathrm{I}_{\mathbb{R}^-}(X)$ and $Y_b = \mathrm{I}_{\mathbb{R}^-}(Y)$, where I is the indicator function. Clearly, the random variable X_b has a Bernoulli distribution with parameter $\frac{1}{2} + C_w$, where $C_w = \frac{1-2w}{2}\mathrm{erf}\left(\frac{-\mu}{\sqrt{2}\sigma}\right)$. The random variable Y_b has a Bernoulli distribution with parameter $(1-\bar{p})(0.5(1+\mathrm{erf}(\frac{\bar{\mu}_1}{\sqrt{2}\bar{\sigma}_1}))) + (\bar{p})(0.5(1+\mathrm{erf}(\frac{\bar{\mu}_2}{\sqrt{2}\bar{\sigma}_2})))$. Then by using the data processing inequality, we have

$$\mathrm{KL}\left(G^*, G'(\bar{\theta})\right) \geq \mathrm{KL}\left(\mathcal{B}(0.5 + C_w), \mathcal{B}\left((1-\bar{p})(0.5(1+\mathrm{erf}(\frac{\bar{\mu}_1}{\sqrt{2}\bar{\sigma}_1}))) + (\bar{p})(0.5(1+\mathrm{erf}(\frac{\bar{\mu}_2}{\sqrt{2}\bar{\sigma}_2})))\right)\right) \tag{14}$$

Next using proposition 1, we can write

$$\mathrm{KL}\left(G^*, G'(\bar{\theta})\right) \geq 2\left(C_w - 0.5(\bar{p}\mathrm{erf}(\frac{\bar{\mu}_2}{\sqrt{2}\bar{\sigma}_2}) + (1-\bar{p})\mathrm{erf}(\frac{\bar{\mu}_1}{\sqrt{2}\bar{\sigma}_1}))\right)^2 \tag{15}$$

Combining this lower bound with the upper bound obtained in equation 11, we have

$$2\left(-\sqrt{\frac{C_2}{2}} + C_w\right) \leq \tau(\bar{\theta}) \leq 2\left(\sqrt{\frac{C_2}{2}} + C_w\right) \tag{16}$$

where, $\tau(\bar{\theta}) := \left((\bar{p})\mathrm{erf}(\frac{\bar{\mu}_2}{\sqrt{2}\bar{\sigma}_2}) + (1-\bar{p})\mathrm{erf}(\frac{\bar{\mu}_1}{\sqrt{2}\bar{\sigma}_1})\right)$

Alternately, we can also use proposition 2 by noting that total variation distance of two Bernoulli distributions $\mathbb{P}, \mathbb{Q}$ with parameters p, q is $|p-q|$, and hence proposition 2 leads to the following inequality:

$$-\log(1-(p-q)^2) \leq \mathrm{KL}(\mathbb{P}, \mathbb{Q}). \tag{17}$$

Applying this inequality in (14) and (11), we have

$$C_2 \geq \mathrm{KL}\left(G^*, G'(\bar{\theta})\right) \geq -\log\left(1 - \left(\frac{\tau(\bar{\theta})}{2} - C_w\right)^2\right) \tag{18}$$

This can be rewritten as

$$2\left(-\sqrt{1-e^{-C_2}} + C_w\right) \leq \tau(\bar{\theta}) \leq 2\left(\sqrt{1-e^{-C_2}} + C_w\right) \tag{19}$$

Next we empirically compare the bounds obtained using approach 1 and approach 2 for different (μ, σ, π) for varying values of t. For a given value of t, we simulate 200 datasets and pick the maximum of $\tau(\bar{\theta}) = \left|\left(\bar{p}\mathrm{erf}(\frac{\bar{\mu}_2}{\sqrt{2}\bar{\sigma}_2}) + (1-\bar{p})\mathrm{erf}(\frac{\bar{\mu}_1}{\sqrt{2}\bar{\sigma}_1})\right)\right|$ among these 200 output solutions. From figure 8, we see that the bound is most effective when the separation of the components in the true distribution is low and proportion of the contaminating distribution is high. Also, we note that approach -1 gives a tighter bound for most values of b and hence we go with it in our theorem 1.

It is possible to generalise Theorem 1 to the multivariate case. Here we have considered this particular univariate setup because of the availability of closed form expressions of KL-divergences. For general settings, using this same approach, it is possible to compute similar bounds if the closed form expressions for KL-divergences are available.

[3] https://github.com/ccanonne/probabilitydistributiontoolbox/blob/master/pinskers-and-beyond.pdf

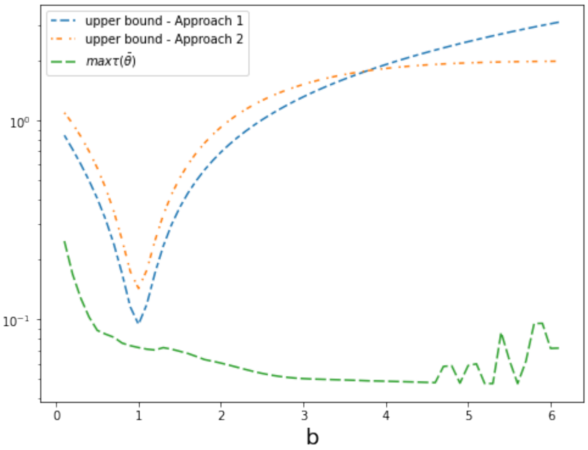

(a) $\mu = 2.0, \sigma = 20, \pi = 0.35$

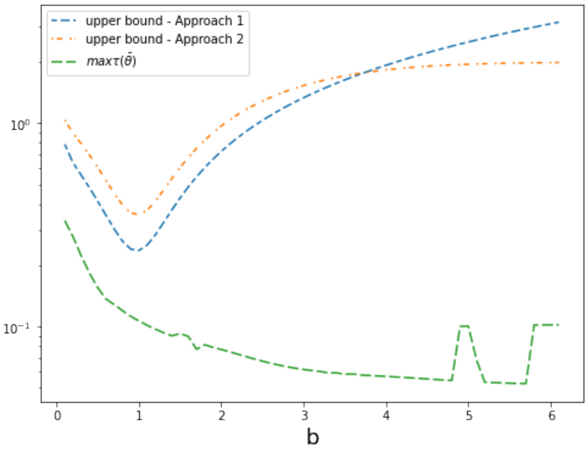

(b) $\mu = 5.0, \sigma = 20, \pi = 0.35$

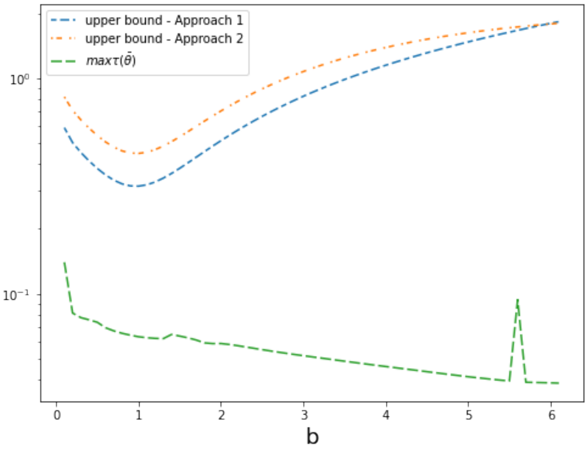

(c) $\mu = 5.0, \sigma = 20, \pi = 0.45$

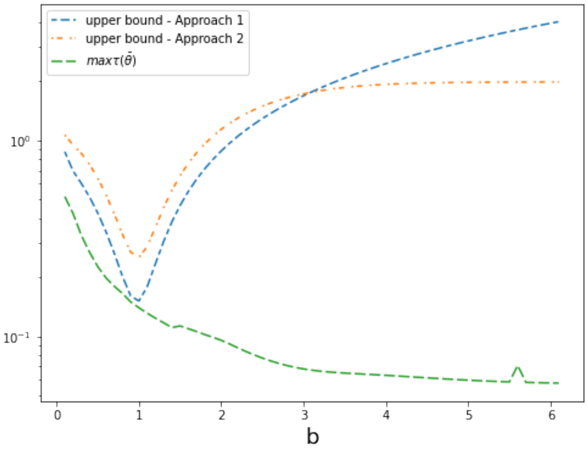

(d) $\mu = 5.0, \sigma = 20, \pi = 0.25$

Figure 8. Empirical comparison of the bounds for different (μ, σ, π) for varying values of b

G.2 Proof of Theorem 2

First, we show the boundedness for the simple case of a univariate mixture model as it is straightforward to follow and later proceed to the proof of the multivariate case.

Proof. **Univariate:** With loss of generality, let the component 1 - $\mathcal{N}(x; \mu_1, \sigma_1^2)$ - is the one that is fitting over a single point and reducing its variance $\hat{\sigma}_1^2 \to 0$; also, assume that $\hat{\sigma}_1^2 \leq \hat{\sigma}_2^2 \leq c < \infty$

First note that the value of likelihood satisfies the following inequality:

$$\mathcal{N}(x; \mu, \sigma^2) \leq \frac{1}{\sqrt{2\pi}\sigma} \tag{20}$$

For one-dimensional case, the penalized likelihood of SIA for an observation x_i can be written as follows:

$$\log(\hat{\pi}_1\mathcal{N}(x_i;\hat{\mu}_1,\hat{\sigma}_1^2)+\hat{\pi}_2\mathcal{N}(x_i;\hat{\mu}_2,\hat{\sigma}_2^2)) - w_1\left(\log\frac{\hat{\sigma}_2}{\hat{\sigma}_1}+\frac{\hat{\sigma}_1^2+(\hat{\mu}_1-\hat{\mu}_2)^2}{2\hat{\sigma}_2^2}-\frac{1}{2}\right) - w_2\left(\log\frac{\hat{\sigma}_1}{\hat{\sigma}_2}+\frac{\hat{\sigma}_2^2+(\hat{\mu}_1-\hat{\mu}_2)^2}{2\hat{\sigma}_1^2}-\frac{1}{2}\right) \tag{21}$$

(By using eq 20 and setting $(w_3 = w_2(\hat{\sigma}_2^2+(\hat{\mu}_1-\hat{\mu}_2)^2))$, and leaving out -ve terms)

$$\le \log(\frac{\hat{\pi}_1}{\hat{\sigma}_1}+\frac{\hat{\pi}_2}{\hat{\sigma}_2}) - w_2\log\hat{\sigma}_1 - \frac{w_3}{2\hat{\sigma}_1^2} - \log\sqrt{2\pi}+w_2\log c \tag{22}$$

$$\le \log(\frac{\hat{\pi}_1+\hat{\pi}_2}{\hat{\sigma}_1}) - w_2\log\hat{\sigma}_1 - \frac{w_3}{2\hat{\sigma}_1^2} + w_2\log c(\text{where } w_4 = 1+w_2)$$

$$\le -w_4\log\hat{\sigma}_1 - \frac{w_3}{2\hat{\sigma}_1^2} + w_2\log c \tag{23}$$

(Differentiating wrt σ_1 and setting to 0, we find $\sigma_1^* = \sqrt{\frac{w_3}{w_4}}$)

$$\le -w_4\log\sqrt{\frac{w_3}{w_4}} - 0.5w_4 + w_2\log c \tag{24}$$

The terms in equation 24 are all constants hence the SIA likelihood is bounded. It should be noted that since we started with the assumption that only the first component becomes degenerate (i.e. $\sigma_1^2 \to 0$) while the second component doesn't, the same boundedness result can be trivially generalized to the case where there are more than 2 components.

Multivariate: $\lambda_1(\hat{\Sigma}_1), \lambda_1(\hat{\Sigma}_2) \le c_1$ where $\lambda_p(.)$ and $\lambda_1(.)$ denotes the smallest and the largest eigenvalue of the $\mathbb{R}^{p\times p}$ matrix. The likelihood can be made unbounded by making the determinant of $\hat{\Sigma}_1$ close to zero i.e. $|\hat{\Sigma}_1| \to 0$.

First, we bound the following terms:

$$0.5w_2\log(|\hat{\Sigma}_2|) \le 0.5w_2 p\log(c_1) = C(\text{some constant}) \tag{25}$$

$$\log(\hat{\pi}_1\mathcal{N}(x_i;\hat{\mu}_1,\hat{\Sigma}_1)+\hat{\pi}_2\mathcal{N}(x_i;\hat{\mu}_2,\hat{\Sigma}_2)) - 0.5*w_1\left(\log\frac{|\hat{\Sigma}_2|}{|\hat{\Sigma}_1|}+\text{tr}(\hat{\Sigma}_2^{-1}\hat{\Sigma}_1)+(\mu_2-\mu_1)^T\hat{\Sigma}_2^{-1}(\mu_2-\mu_1)^T\right)$$

$$-0.5*w_2\left(\log\frac{|\hat{\Sigma}_1|}{|\hat{\Sigma}_2|}+\text{tr}(\hat{\Sigma}_1^{-1}\hat{\Sigma}_2)+(\mu_2-\mu_1)^T\hat{\Sigma}_1^{-1}(\mu_2-\mu_1)^T\right) \tag{26}$$

(By using eq 20 and 25, and leaving out -ve terms and absorbing the constants into C)

$$\le 0.5*\log(\frac{\hat{\pi}_1}{|\hat{\Sigma}_1|}+\frac{\hat{\pi}_2}{|\hat{\Sigma}_2|}) - 0.5*w_2\log(|\hat{\Sigma}_1|) - 0.5*w_2\text{tr}(\hat{\Sigma}_1^{-1}\hat{\Sigma}_2) \tag{27}$$

$$= 0.5*\left(\log(\frac{\hat{\pi}_1+\hat{\pi}_2}{|\hat{\Sigma}_1|}) - w_2\log(|\hat{\Sigma}_1|) - w_2\text{tr}(\hat{\Sigma}_1^{-1}\hat{\Sigma}_2)\right) \tag{28}$$

$$= 0.5*\left(-\log(|\hat{\Sigma}_1|) - w_2\log(|\hat{\Sigma}_1|) - w_2\text{tr}(\hat{\Sigma}_1^{-1}\hat{\Sigma}_2)\right) \tag{29}$$

$$= 0.5*\left(-w_3\log(|\hat{\Sigma}_1|) - w_2\text{tr}(\hat{\Sigma}_1^{-1}\hat{\Sigma}_2)\right) \quad (\text{where } w_3 = 1+w_2) \tag{30}$$

$$= 0.5*\left(w_3\log(|\hat{\Sigma}_1^{-1}|) - w_2\text{tr}(\hat{\Sigma}_1^{-1}\hat{\Sigma}_2)\right) \tag{31}$$

Note that equation 31 is concave in Σ_1^{-1} as $\log(|.|)$ is a concave function and trace is affine [28]; hence, if a maximum exists, it is the global maximum. We can find the maximum by differentiating with respect to Σ_1^{-1} and setting to zero;

$$\nabla_{\Sigma_1^{-1}}\left(w_3\log(|\hat{\Sigma}_1^{-1}|) - w_2\text{tr}(\hat{\Sigma}_1^{-1}\hat{\Sigma}_2)\right) = w_3\hat{\Sigma}_1 - w_2\hat{\Sigma}_2 = 0 \tag{32}$$

$$\implies \hat{\Sigma}_1 = \frac{w_2}{w_3}\hat{\Sigma}_2 \tag{33}$$

Therefore, the final upper bound of the log-likelihood is given by

$$-0.5(1+w_2)\left(\log(|\frac{w_2}{1+w_2}\hat{\Sigma}_2|)+p\right)+C \tag{34}$$

□

H Closed Forms

In this section, we illustrate the difficulty of deriving the closed form expressions of the gradients of the penalized likelihood used in SIA. These closed form expressions are required to develop an EM algorithm for inference. Consider the mean update in EM for the unpenalized loglikelihood:

$$\frac{\partial \log \mathcal{L}}{\partial \mu_k} = -\sum_{i=1}^{n} \gamma(z_{ik}) \Sigma_k^{-1}(\mathbf{x}_i - \mu_k) \tag{35}$$

where $\gamma(z_{ik})$ is defined as the responsibility and is equal to $\frac{\pi_k \mathcal{N}(\mathbf{x}_i|\boldsymbol{\mu}_k,\boldsymbol{\Sigma}_k)}{\sum_{j=1}^{K}\pi_j \mathcal{N}(\mathbf{x}_i|\boldsymbol{\mu}_j,\boldsymbol{\Sigma}_j)}$. Setting $\frac{\partial \log \mathcal{L}}{\partial \mu_k}$ to zero, we obtain $\mu_k = \frac{\sum_{i=1}^{n}\gamma(z_{ik})\mathbf{x}_i}{\sum_{i=1}^{n}\gamma(z_{ik})}$. Note that the computation of the mean update μ_k can be easily parallelized in the M-step because it does not involve other mean terms μ_j where $j \neq k$.

Now, consider the gradient of the penalized loglikelihood:

$$\frac{\partial \log(\mathcal{L} - w_1 \times KLF - w_2 \times KLB)}{\partial \mu_k} = -\sum_{i=1}^{n}\gamma(z_{ik})\Sigma_k^{-1}(\mathbf{x}_i - \mu_k) - w_1 \sum_{j\neq k} 2\Sigma_j^{-1}(\mu_k - \mu_j) - w_2 \sum_{j \neq k} 2\Sigma_k^{-1}(\mu_k - \mu_j) \tag{36}$$

Setting the above expression to zero, we can obtain the expression for the mean μ_k as:

$$\begin{aligned}\mu_k = &\left(\sum_{i=1}^{n}\gamma(z_{ik})\Sigma_k^{-1} - (w_1+w_2)(\sum_{j\neq k}\left(\Sigma_j^{-1}+\Sigma_k^{-1}\right))\right)^{-1} \\ &\left(\sum_{i=1}^{n}\gamma(z_{ik})\Sigma_k^{-1}\mathbf{x}_i - (w_1+w_2)(\sum_{j\neq k}\left(\Sigma_j^{-1}+\Sigma_k^{-1}\right)\mu_j)\right)\end{aligned} \tag{37}$$

As seen above, even though the process of obtaining the updates is straightforward, it is laborious. For the update of covariance estimates, we could not find a closed form estimate for the penalized loglikelihood. Further, note the mean update requires knowledge of other mean terms μ_j and hence cannot be parallelized. EM based updates for the more flexible models such as MFA will be even more cumbersome.

In contrast, using Gradient Descent (GD) based inference using Automatic Differentiation (AD) avoids the need to compute such laborious gradient updates by hand or to hard code them in the software program. AD-GD based inference approach is model-agnostic as AD software are blackbox tools where we just need to input the loglikelihood; they don't require closed forms of the gradients. Thus the same software implementation can be easily extended for more flexible models such as MFA, PGMM etc. This is not the case with the traditional EM based inference where the updates have to be derived and re-implemented for each model.

I Likelihood Surface Visualization

To understand how the penalization in (1) helps in clustering we visualize the log-likelihood surface using the technique of [29] for neural network loss landscapes. Given two sets of parameters θ_1, θ_2, the surface function $S(\alpha,\beta) = \mathcal{L}(\alpha\theta_1 + \beta\theta_2)$ where $0 \leq \alpha, \beta \leq 1$ is used to analyze the log-likelihood $\mathcal{L}$.

As an illustration, we use the Pinwheel data described in section 3 with the parameters obtained for (g) and (a) of fig. 1 as θ_1 and θ_2 respectively and plot the log-likelihood and penalized log-likelihood in fig. 9 (a) and (b) respectively. It is evident in fig. 9 (a) that the log-likelihood has a significant plateau region which has almost similar (unpenalized) likelihood. However, for SIA likelihood in (b), such a plateau does not exist and there exists a clear maxima at $(\alpha = 1, \beta = 0)$. As expected θ_1 ((g) of fig. 1) has a better clustering output, despite having similar (unpenalized) likelihood as that of θ_2.

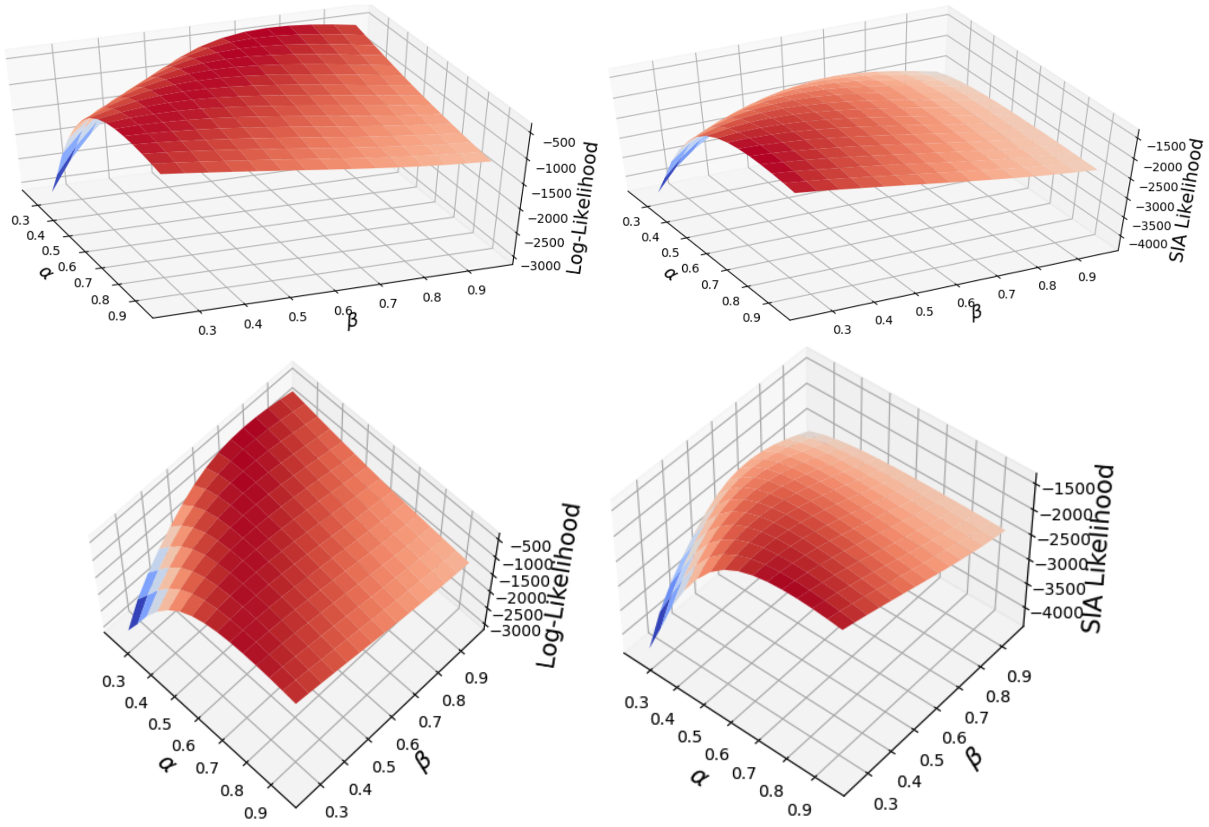

Figure 9. (Left:) Side-view and Top-view of GMM Log-likelihood show a plateau region ; (Right:) Side-view and Top-view of Penalized log-likelihood has no plateau and a clear peak corresponding to good clustering.

J Wallclock time

We benchmark the runtime of SIA with respect to AD-GD and EM. We do so by simulating 50 datasets, each containing 100 datapoints sampled randomly, for each setting. We run all the algorithms to convergence in likelihood (tolerance 1e-5) or a maximum of 50 iterations, which ever happens earlier. We evaluate with the number of clusters $K = \{3, 5\}$ and data dimensions $p = \{2, 5, 10, 50\}$. All experiments were run using Autograd, Numpy and Sklearn packages in Python 3.7 on Dell Windows 10 machine (Intel i7-6700 quadcore CPU@3.40GHz; 8 GB RAM; 500 GB HDD).

Table 20 shows the runtime of all the algorithms. We observed that with increasing dimensionality, the number of iterations for EM reduced, and at $p = 50$, EM failed to go beyond iteration 1. As expected, SIA takes roughly double the time taken by AD-GD.

Table 20. Average Runtime in seconds with data dimensions $2, 5, 10, 50$.

# of clusters	Algorithm	2	5	10	50
3	SIA	7.900	7.926	8.037	11.508
3	AD-GD	4.471	4.467	4.529	6.50
3	EM	0.009	0.006	0.009	-
5	SIA	20.571	20.785	21.238	30.041
5	AD-GD	11.127	11.194	11.433	16.167
5	EM	0.036	0.025	0.014	-

K Illustration of unbalanced clusters on Pinwheel data

We illustrate the effect of unbalanced clusters on the same pinwheel data that is discussed in section 3. In the first experiment, instead of (100,100,100) number of points respectively in the three clusters, we have (100,50,20) number of points respectively for the three clusters. Please refer to fig. 10 (a) and (b) for the clustering results with SIA with this dataset. In the second experiment we have (100,50,50) number of points. Please refer to fig. 10 (c) and (d) for the results obtained from SIA.

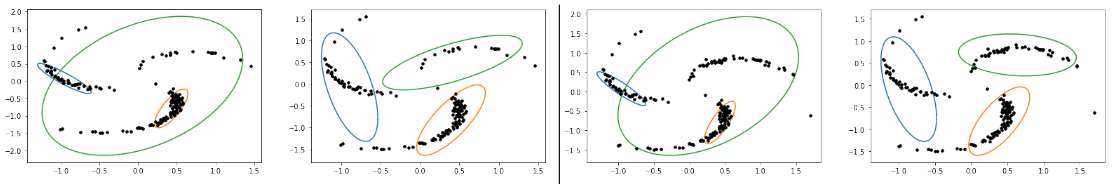

(a) After Step 1: ARI = 0.602 MPKL = 220.887 Loglikelihood = -139.4

(b) After Step 2: ARI = 0.804 MPKL = 41.77, Loglikelihood = -191.9

(c) After Step 1: ARI = 0.606 MPKL = 279.769, Loglikelihood = -228.2

(d) After Step 2: ARI =0.847 MPKL = 39.251, Loglikelihood = -261.52

Figure 10. Clustering using SIA for unbalanced pinwheel data. Componentwise number of datapoints: $\{100, 50, 20\}$ (a and b), $\{100, 50, 50\}$ (c and d).

L Additional Experiments

L.1 Performance of SIA

We evaluate the performance of SIA in cases of under-specification and over-specification of number of components. For these experiments, introducing misspecification by cubing the data points (as done in simulations in section 5) lead to mixing of the components and does not allow us to study the effects of over–/under-specification of components. So, we introduce misspecification through contamination by t-distribution as described below.

L.1.1 Under-specified number of components

We sample 120 datapoints from 3-component p-dimensional GMMs (40 datapoints from each component) with spherical covariances. The means of the components are $(0, \ldots, 0)_p$, $(-1, \ldots, -1)_p$ and $(1, \ldots, 1)_p$. In addition, we sample 30 datapoints from 3 component p-dimensional t-distribution mixture (10 datapoints from each component), with the same means and covariances, with two degrees of freedom. Thus, there are 150 datapoints sampled from a contaminated GMM. The dimensionality of p is chosen from $\{2, 3, 5, 7, 10\}$. We fit a *misspecified* 2-component GMM over these 150 datapoints. For each setting, we simulated 50 datasets and compute ARI of the clustering output. The results are given in Table 21. We find that both EM and MClust do not perform well when compared to AD-GD and SIA.

L.1.2 Over-specified number of components

We sample 80 datapoints from 2 component p-dimensional GMMs (40 datapoints from each component) with spherical covariances. The means of the components are $(0, \ldots, 0)_p$ and $(1, \ldots, 1)_p$. In addition, we sample 20 datapoints from 2 component p-dimensional t-distribution mixture, with the same means and covariances, with three degrees of freedom. Thus, the overall 100 datapoints are sampled from a contaminated GMM. The dimensionality of p is chosen from $\{2, 3, 5, 7, 10\}$. We fit a *misspecified* 3-component GMM over these 100 datapoints. For each setting, we simulated 50 datasets and compute ARI of the clustering output. The results are given in Table 22.

Table 21. Average (std. dev) ARI with increasing dimensionality (p) for under-specified mixtures

p:	2	3	5	7	10
SIA	**0.240**	**0.279**	**0.330**	**0.306**	**0.328**
	**(0.127)**	**(0.145)**	**(0.158)**	**(0.191)**	**(0.196)**
AD-GD	0.238	0.277	0.323	0.302	0.323
	(0.126)	(0.144)	(0.155)	(0.189)	(0.193)
EM	0.042	0.021	0.066	0.047	0.100
	(0.103)	(0.074)	(0.118)	(0.115)	(0.167)
MClust	0.055	0.046	0.1289	0.092	0.125
	(0.121)	(0.114)	(0.186)	(0.175)	(0.200)

Table 22. Average (std. dev) ARI with increasing dimensionality (p) for over-specified mixtures

p:	2	3	5	7	10
SIA	**0.189**	**0.266**	0.369	0.517	0.614
	**(0.090)**	**(0.124)**	(0.166)	(0.182)	(0.205)
AD-GD	0.182	0.252	0.356	0.501	0.607
	(0.089)	(0.122)	(0.160)	(0.179)	(0.203)
EM	0.059	0.081	0.129	0.147	0.359
	(0.094)	(0.096)	(0.134)	(0.181)	(0.244)
MClust	0.074	0.168	**0.381**	**0.523**	**0.666**
	(0.094)	(0.127)	**(0.132)**	**(0.137)**	**(0.120)**

L.2 Model Selection

In this section we compare the performance of MPKL with that of AIC and BIC, as model selection criteria, on synthetic data. Note that MPKL is a general model selection criterion and can be used with any inference technique and even when misspecification is not suspected.

We follow the setting used in [30, 31] and simulate a 4 component 5-dimensional datasets as shown in Table 23. We assume unit spherical covariance matrices for each component. The parameters of each component across the 5 dimensions are varied as shown in Table 23 – all the components have the same parameters in 2 dimensions (4 and 5), only dimensions 1, 2, 3 are discriminating for components 2,3,4 respectively. The value of λ controls the cluster separation. For each component we sampled ten datapoints. Three different sets of simulated data with varying cluster separation are obtained by choosing the value of λ to be 1, 5 and 10 respectively. This experiment is repeated with 10 different seeds for each value of λ. We use SIA for clustering with each value of $K \in \{3,4,5\}$. We compare the number of clusters selected using AIC, BIC and MPKL.

Table 23. Simulations for Model Selection for 5-dimensional datasets. Along each dimension, each component has unit variance. Means are given below:

Features	C-1	C-2	C-3	C-4
1	0	λ	0	0
2	0	0	λ	0
3	0	0	0	λ
4	0	0	0	0
5	0	0	0	0

Table 24. Number of times (in 10 reps) 3, 4, 5 components are selected with each criterion – MPKL (M), AIC (A), BIC (B) – for a 4-component 5-dimensional dataset with 3 cluster separations ($\lambda = 1, 5, 10$).

λ:	1			5			10		
#clusters	M	A	B	M	A	B	M	A	B
3	0	7	10	0	0	0	0	0	0
4	10	3	0	10	10	10	10	10	10
5	0	0	0	0	0	0	0	0	0

The results are given in Table 24. When the dimensionality is low and cluster separation is small, both AIC and BIC underestimate the number of components. MPKL correctly estimates the number of components in all the cases.

We repeat the above experiment for $p = 50$; details of the experiments are given in Table 25. The parameters of each component across the 50 dimensions are varied as shown in table 25, i.e., all the components have the same parameters for 35 dimensions, only dimensions 1-5, 5-10, 10-15 are discriminating for components 2,3,4 respectively. All other simulation settings are same as described above for the 5-dimensional case.

Table 25. Simulations for Model Selection for 50-dimensional datasets. Along each dimension, each component has unit variance. Means are given below:

Features	C-1	C-2	C-3	C-4
1-5	0	λ	0	0
5-10	0	0	λ	0
10-15	0	0	0	λ
15-50	0	0	0	0

Table 26. Number of times (in 10 reps) 3, 4, 5 components are selected with each criterion – MPKL (M), AIC (A), BIC (B) – for a 4-component 50-dimensional dataset with 3 cluster separations ($\lambda = 1, 5, 10$).

λ:	1			5			10		
#clusters	M	A	B	M	A	B	M	A	B
3	10	10	10	1	10	10	1	0	10
4	0	0	0	7	0	0	8	10	0
5	0	0	0	2	0	0	1	0	0

Table 26 shows that at very low cluster separation ($\lambda = 1$), both the criteria do not select 4 clusters. At moderate and high cluster separation ($\lambda = 5, 10$), BIC underestimates the number of clusters to 3, which is consistent with

previous findings [8]. AIC, on the other hand, performs well only when the cluster separation is very high. MPKL identifies 4 clusters in 7 out of 10 times at moderate separation and 8 out of 10 times at high cluster separation.

M Additional details of Wine Case Study

We give the scatterplot matrix for the recommended model S9 here in Figure 11. The scatterplot for the best model for each value of K is given in Figure 12.

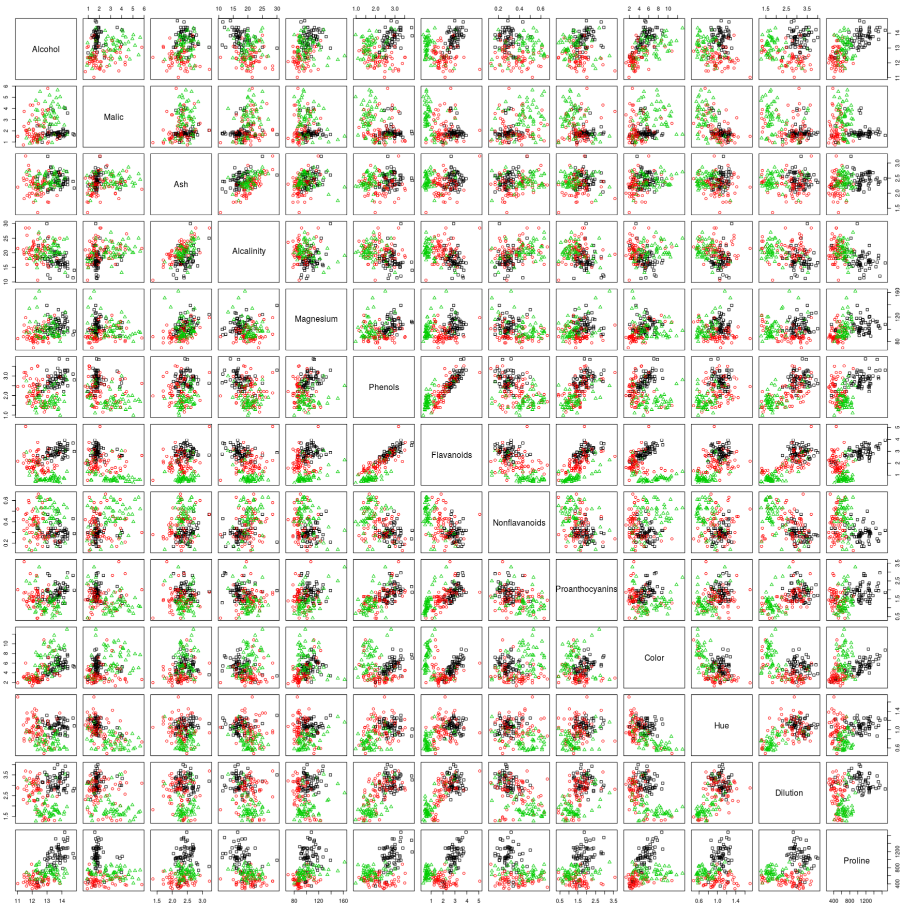

Figure 11. Scatterplot matrix for the recommended model

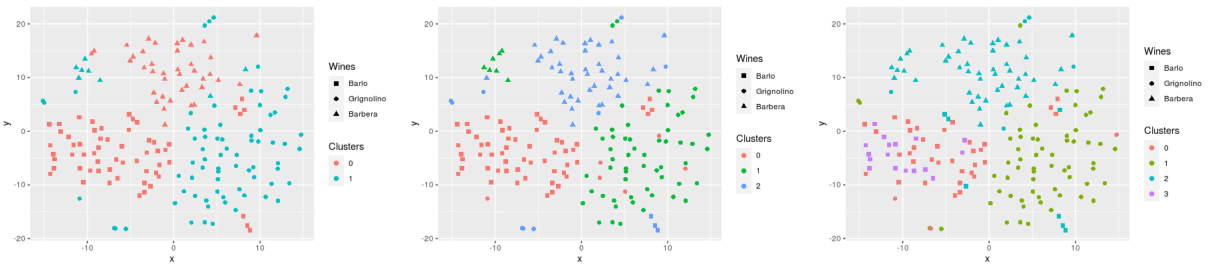

Figure 12. tSNE plots for the best model for each value of K (S. Nos. 3,9 and 13 in Table 12); Clusters obtained for $K = 2$ (above), $K = 3$ (middle) and $K = 4$ (below).

N Generation of Covariance matrices

Consider a covariances matrix $\Sigma = \Sigma^{1/2}(\Sigma^{1/2})^T$. The parameters of $\Sigma^{1/2}$ are sampled randomly from a standard normal distribution to capture different covariance structures in Σ. We illustrate that this random sampling gives different covariance structures by plotting 1000 datapoints sampled from $\mathcal{N}((0,0),\Sigma)$ for the first 10 seeds as shown in Figure 13.

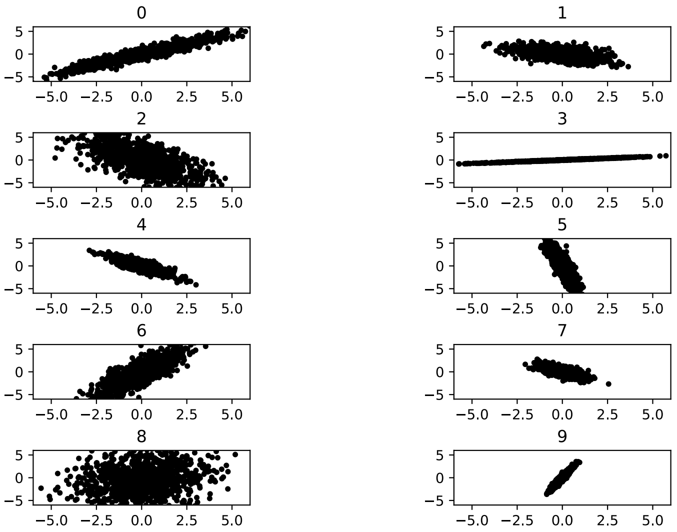

Figure 13. Different seeds give different covariances structures (seed value is mentioned above each subplot).

References

1. McLachlan, G. J. & Peel, D. *Finite Mixture Models* (John Wiley & Sons, New York, 2000).
2. Konishi, S. & Kitagawa, G. *Information criteria and statistical modeling* (Springer Science & Business Media, 2008).
3. Lv, J. & Liu, J. S. Model selection principles in misspecified models. *J. Royal Stat. Soc. Ser. B: Stat. Methodol.* 141–167 (2014).
4. Takeuchi, K. Distribution of informational statistics and a criterion of model fitting. *Suri-Kagaku (Mathematical Sci. Jpn.* **153**, 12–18 (1976).
5. Amari, S.-I., Park, H. & Ozeki, T. Singularities affect dynamics of learning in neuromanifolds. *Neural Comput.* **18**, 1007–1065 (2006).
6. Akaho, S. & Kappen, H. J. Nonmonotonic generalization bias of Gaussian mixture models. *Neural Comput.* **12**, 1411–1427 (2000).
7. Giraud, C. *Introduction to high-dimensional statistics* (Chapman and Hall/CRC, 2014).
8. Melnykov, V., Maitra, R. *et al.* Finite mixture models and model-based clustering. *Stat. Surv.* **4**, 80–116 (2010).
9. Kasa, S. R. & Rajan, V. Improved inference of Gaussian mixture copula model for clustering and reproducibility analysis using automatic differentiation. *Econom. Stat.* **22**, 67–97 (2022). The 2nd Special issue on Mixture Models.
10. Baydin, A. G., Pearlmutter, B. A., Radul, A. A. & Siskind, J. M. Automatic differentiation in machine learning: a survey. *J. Mach. Learn. Res.* **18**, 1–43 (2018).
11. Margossian, C. C. A review of automatic differentiation and its efficient implementation. *Wiley Interdiscip. Rev. Data Min. Knowl. Discov.* **9**, e1305 (2019).
12. Maclaurin, D., Duvenaud, D. & Adams, R. P. Autograd: Effortless gradients in numpy. In *ICML 2015 AutoML Workshop*, vol. 238 (2015).
13. Pav, S. E. Madness: a package for multivariate automatic differentiation (2016).
14. Paszke, A. *et al.* Automatic differentiation in PyTorch (2017).

15. Carpenter, B. *et al.* The Stan math library: Reverse-mode automatic differentiation in C++. *arXiv preprint arXiv:1509.07164* (2015).

16. Kingma, D. P. & Ba, J. Adam: a method for stochastic optimization. *Proc. 3rd Int. Conf. on Learn. Represent. (ICLR)* **1412** (2015).

17. Salakhutdinov, R., Roweis, S. T. & Ghahramani, Z. Optimization with EM and expectation-conjugate-gradient. In *Proceedings of the 20th International Conference on Machine Learning (ICML-03)*, 672–679 (2003).

18. Robert, C. *Machine learning, a probabilistic perspective* (Taylor & Francis, 2014).

19. Alexandrovich, G. An exact Newton's method for ML estimation of a Gaussian mixture (2014).

20. Punzo, A. & McNicholas, P. D. Parsimonious mixtures of multivariate contaminated normal distributions. *Biom. J.* **58**, 1506–1537 (2016).

21. Dwivedi, R., Khamaru, K., Wainwright, M. J., Jordan, M. I. *et al.* Theoretical guarantees for EM under misspecified Gaussian mixture models. In *Advances in Neural Information Processing Systems*, 9681–9689 (2018).

22. Rajan, V. & Bhattacharya, S. Dependency clustering of mixed data with Gaussian mixture copulas. In *IJCAI*, 1967–1973 (2016).

23. Kasa, S. R., Bhattacharya, S. & Rajan, V. Gaussian mixture copulas for high-dimensional clustering and dependency-based subtyping. *Bioinformatics* (2019).

24. Nguyen, X. *et al.* Convergence of latent mixing measures in finite and infinite mixture models. *The Annals Stat.* **41**, 370–400 (2013).

25. Pinsker, M. S. *Information and information stability of random variables and processes.* Translated and edited by Amiel Feinstein (Holden-Day, Inc., San Francisco, Calif.-London-Amsterdam, 1964).

26. Lattimore, T. & Szepesvári, Cs. *Bandit algorithms* (Cambridge U. Press, 2020).

27. Bretagnolle, J. & Huber, C. Estimation des densités: risque minimax. In *Séminaire de Probabilités, XII (Univ. Strasbourg, Strasbourg, 1976/1977)*, vol. 649 of *Lecture Notes in Math.*, 342–363 (Springer, Berlin, 1978).

28. Boyd, S. & Vandenberghe, L. *Convex optimization* (Cambridge University Press, 2004).

29. Li, H., Xu, Z., Taylor, G., Studer, C. & Goldstein, T. Visualizing the loss landscape of neural nets. In *Advances in Neural Information Processing Systems*, 6389–6399 (2018).

30. Pan, W. & Shen, X. Penalized model-based clustering with application to variable selection. *J. Mach. Learn. Res.* **8**, 1145–1164 (2007).

31. Guo, J., Levina, E., Michailidis, G. & Zhu, J. Pairwise variable selection for high-dimensional model-based clustering. *Biometrics* **66**, 793–804 (2010).
